# No Ancient DNA Damage in Actinobacteria from the Neanderthal Bone

**DOI:** 10.1371/journal.pone.0062799

**Published:** 2013-05-03

**Authors:** Katarzyna Zaremba-Niedźwiedzka, Siv G. E. Andersson

**Affiliations:** Department of Molecular Evolution, Cell and Molecular Biology, Science for Life Laboratory, Biomedical Centre, Uppsala University, Uppsala, Sweden; Baylor College of Medicine, United States of America

## Abstract

**Background:**

The Neanderthal genome was recently sequenced using DNA extracted from a 38,000-year-old fossil. At the start of the project, the fraction of mammalian and bacterial DNA in the sample was estimated to be <6% and 9%, respectively. Treatment with restriction enzymes prior to sequencing increased the relative proportion of mammalian DNA to 15%, but the large majority of sequences remain uncharacterized.

**Principal Findings:**

Our taxonomic profiling of 3.95 Gb of Neanderthal DNA isolated from the Vindija Neanderthal Vi33.16 fossil showed that 90% of about 50,000 rRNA gene sequence reads were of bacterial origin, of which Actinobacteria accounted for more than 75%. Actinobacteria also represented more than 80% of the PCR-amplified 16S rRNA gene sequences from a cave sediment sample taken from the same G layer as the Neanderthal bone. However, phylogenetic analyses did not identify any sediment clones that were closely related to the bone-derived sequences. We analysed the patterns of nucleotide differences in the individual sequence reads compared to the assembled consensus sequences of the rRNA gene sequences. The typical ancient nucleotide substitution pattern with a majority of C to T changes indicative of DNA damage was observed for the Neanderthal rRNA gene sequences, but not for the Streptomyces-like rRNA gene sequences.

**Conclusions/Significance:**

Our analyses suggest that the Actinobacteria, and especially members of the Streptomycetales, contribute the majority of sequences in the DNA extracted from the Neanderthal fossil Vi33.16. The bacterial DNA showed no signs of damage, and we hypothesize that it was derived from bacteria that have been enriched inside the bone. The bioinformatic approach used here paves the way for future studies of microbial compositions and patterns of DNA damage in bacteria from archaeological bones. Such studies can help identify targeted measures to increase the relative amount of endogenous DNA in the sample.

## Introduction

The new developments in sequencing technologies have enabled analyses of mitochondrial and nuclear genomes of ancient organisms that lived thousands of years ago. Using these technologies, draft genome sequences have been assembled from short DNA fragments extracted from bone specimens of a 38,000-year-old Neanderthal found at Vindija Cave in Croatia [1], [2]. Bone fragments and hair have also been used as a source of DNA for the assembly of the genomes of 28,000 and 43,000 years old mammoths discovered in the Taimyr permafrost and in the Bolshaya Kolopatkaya river in Russia [3], [4], [5]. Recently, a draft genome was assembled from DNA extracted from the bone of a 5,300-year-old corpse discovered on the Tisenjoch Pass in the Italian part of the Ötztal Alps and referred to as the Tyrolean Iceman [6].

The preservation of the ancient DNA varies greatly in these samples, depending on many factors including the age and nature of the specimen as well as on the temperature and composition of the surrounding environment. Typically, the content of ancient DNA is only a few percent, although some permafrost-preserved specimens can contain up to 90% of endogenous DNA [1], [3]. Thus, the sequence data collected from these samples does not only contain DNA from the organism of interest, but also DNA from other sources, in varying quantities. These sequences could be derived from microbial contamination during the handling of the fossil, ancestrally present microbes or from microbes involved in the taphonomic process.

Despite attempts to increase the fraction of endogenous DNA by treating the samples with restriction enzymes that target GC-rich bacterial sequences prior to sequencing, most of the sequenced DNA in Neanderthal sample could not be affiliated with any currently known species [2]. This could be because the colonizers of the bone represent uncultivated bacteria for which no genome is yet sequenced, or because the commonly used BLASTn methods are not suitable for the assignment of short (<100 bp) reads, leaving a large majority of reads unassigned. Of the small fraction classified, the estimates of bacterial reads range from 1% in the bones of the Tyrolean Iceman to 9% in the Neanderthal bones and 15% in the woolly mammoth [1], [3], [5], [6]. Actinomycetales is reported to be the most populous order in the Neanderthal bone, representing 6.8% of the total reads. It is hypothesized that the microbial reads are derived bacteria that colonized the bone after the death of the Neanderthal [1], while the *Borrelia*-like sequences identified in the Iceman sample were considered to represent ancient bacteria that colonized the Iceman while he lived [6].

Because it is of general interest to learn more about ancient and modern bacteria that colonize, and possibly degrade, bone material, we have here undertaken a study of the large fraction of previously unknown sequences in the Neanderthal metagenome projects. To this end, we have reanalysed a fraction of the Neanderthal metagenome dataset, with a focus on the bacterial DNA present in the sample. We have also investigated substitution patterns and searched for enzymes putatively involved in the decomposition of the bone. In doing so, we aimed to reconstruct a community profile of the organisms living in the bone of a Neanderthal and set up an approach that can be used for taxonomic profiling of the microbial DNA in ancient DNA samples whether its origin is ancient or not.

## Results

### Data Sets and Work Flow

#### Data sets

Our starting data set was 54 million reads of 454 sequence data from one Vindija Neanderthal Vi33.16 fossil (Table S1). For comparison, we included 30 and 85 million reads from sequencing runs in which the extracted DNA had been digested with two different mixtures of restriction enzymes (Mix1 and Mix2) that cut at GC-rich sequences [2]. The aim of this treatment was to eliminate GC-rich bacterial sequence fragments that contain these restriction sites and thereby increase the otherwise low proportion of Neanderthal DNA.

**Figure 1:**
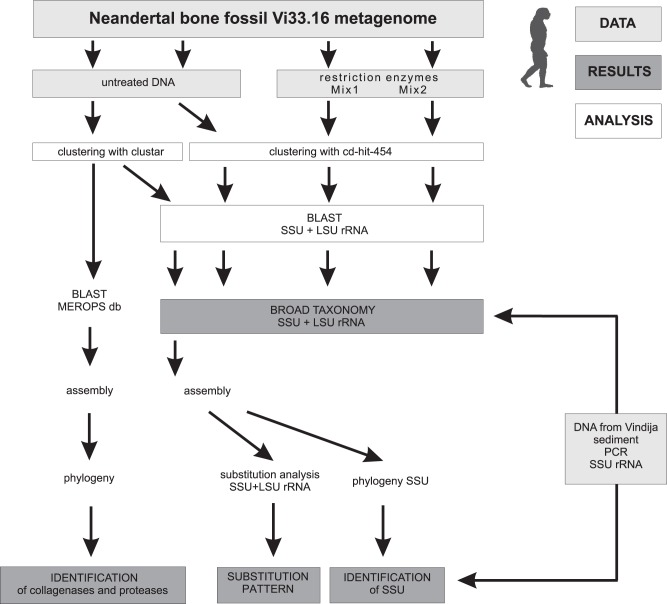
Schematic overview of the analysis workflow.

We removed artificially duplicated reads using a standard metagenomic clustering method (cd-hit-454), which resulted in three datasets of 4.83 Gb (untreated sample), 1.99 Gb (Mix 1) and 4.75 Gb (Mix 2) (Table S1). These datasets were used for broad analyses of taxonomic composition patterns (Figure 1). In parallel, we removed duplicated reads from the untreated sample using a slower, but more strict, clustering procedure (clustar) (Table S2) that was developed for the analyses of this particular dataset [1]. This clustering method reduced the sequence data in the untreated sample to 39.5 million reads and 3.95 Gb. This dataset consisted of a large fraction of short reads with a peak size of 44 bp and a smaller fraction of long sequence reads with a peak size of 255 bp (Figure S1). We used the smaller clustar dataset to examine the phylogenetic placements of the most abundant bacterial taxa, the nucleotide substitution patterns and the possible role of the bacteria inhabiting the Neanderthal bone, as schematically depicted in Figure 1.

#### Testing the performance of BLAST searches of short sequence reads to microbial genomes databases

To evaluate the behavior of short DNA sequences using BLAST analyses, we used a whole-genome 454 sequence data set comprising circa 300,000 reads with a read length of ∼100 bp derived from the ∼1.5 Mb genome of the alphaproteobacterium *Bartonella bovis*, here called Bbovis. We checked the sensitivity of taxonomic assignments for these short reads using BLAST searches against a database that included more than 2,000 microbial genomes of which more than 100 were from the Alphaproteobacteria, including 9 *Bartonella* genomes with an overall sequence identity to *B. bovis* of about 80%. Homologous sequences for about 90% of the *B. bovis* genome could be identified in the other *Bartonella* genomes, so the expectation was that at least 90% of the reads should yield significant hits in these searches. However, only 61% of the *B. bovis* reads gave a hit in BLASTn searches against this database (E<e^−3^). When the *Bartonella* genomes were removed from the microbial database, the fraction of hits was reduced to only 12%, of which 47% were correctly classified as Alphaproteobacteria (Table S3). In a more stringent search (E<e^−10^), only 2% of the reads produced significant hits to sequences in the database, of which 63% were correctly classified as Alphaproteobacteria. We conclude that the performance of BLASTn searches with queries consisting of 100 bp long sequence reads is very poor unless very closely related genomes are present in the databases.

#### Testing the performance of BLAST searches of short sequence reads to rRNA gene sequence database

Next, we asked whether searches against designated rRNA gene sequence databases would be a better approach for automatic, large-scale taxonomic classification schemes. In this test we used the SILVA Ref111 rRNA sequence database, which we manually curated to remove problematic entries, such as sequences containing tRNA genes or metagenomic sequences of unknown origin (eSILVA). In the tests, we used the first hit for assignment (Table S3). As expected, only a small fraction of reads yielded hits to the rRNA gene sequence database (0.05%), of which 95% to 96% were correctly identified as Alphaproteobacteria even if all *Bartonella* sequences had been removed from the database. The median E-values of the hits were high (e^−43^ to e^−52^) and the fraction of hits was constant over a range of E-value cutoff values (e^−3^ to e^−10^).

To further inspect the performance of the eSILVA search we calculated the sensitivity and the specificity (Table S4). In this analysis, true positives were defined as hits overlapping the SSU and LSU rRNA genes with at least 50 bp, while false positives were defined as those mapping outside the rRNA operons and a 1 kb flanking sequence on both sides. Reads mapping to the borders of the gene or within the neighboring 1 kb were ignored for the calculations. The performance of the searches against the eSILVA database was extremely good with a recall of 96–99%, meaning that less than 4% of the hits were false positives. Moreover, a sensitivity of 92–94% showed that less than 8% of the rRNA sequence reads were missed in the analysis. To further improve the robustness of the assignment, we implemented a lowest common ancestor procedure, taking all hits with the same best e-value and making a majority-based assignment with the exclusion of potential single misclassified hits as described in the Methods.

### Taxonomic Compositions

#### The neanderthal sample

**Figure 2:**
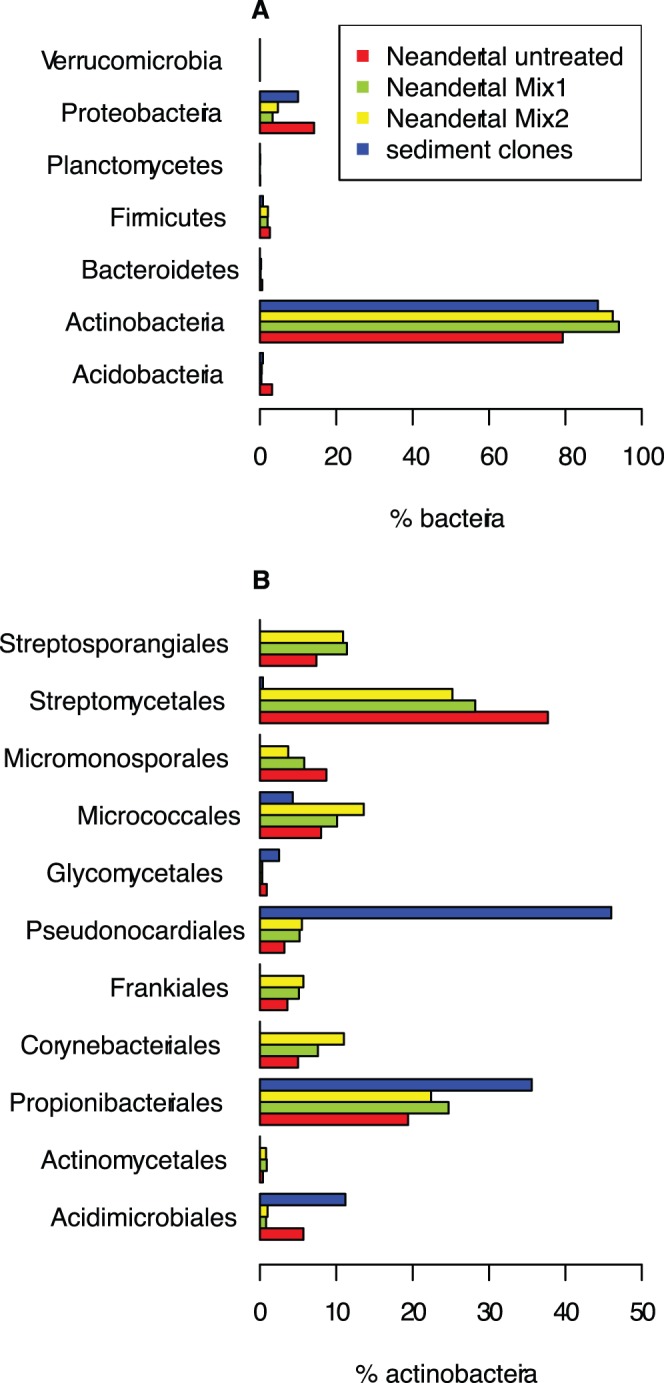
Taxonomic assignments of rRNA gene reads from the Neanderthal and the cave sediment samples. Bacterial community composition patterns at (A) the phylum level and (B) within Actinobacteria. The Neanderthal rRNA gene sequence reads were classified based on BLASTn searches to the SSURef111NR database in SILVA, modified as described in the methods section. The cave sediment rRNA gene sequence reads were classified based on a maximum likelihood phylogeny that included related reference sequences. Bacterial composition patterns at the phylum level were inferred from PCR-amplifications of the bacterial DNA extracted from the cave sediment using (A) universal primers and (B) actinobacterial-specific primers. Scales refer to % of all (A) Bacteria and (B) Actinobacteria.

We used the modified rRNA database search approach outlined above for the taxonomic analyses of the fossil metagenome data sets. Thus, broad taxonomic assignments of both the untreated and the treated Neanderthal data sets were inferred from BLASTn searches (E<10^−10^) against the eSILVA rRNA gene sequence database. Using this procedure, we identified from 48,000 to 140,000 rRNA gene reads per sample, of which 89% to 96% were classified as Bacteria, less than 10% as Eukaryota and less than 2% as Archaea (Table S5). Within bacteria, the diversity at the phylum level was low, with a large majority of the bacterial sequence reads, 74%–95%, being assigned to the Actinobacteria (Figure 2A, Table S6). Proteobacteria accounted for another 3%–14% and represented the second most abundant group (Table S6). Finally, within the Actinobacteria, the majority of sequence reads, 20%–35% were assigned to the Streptomycetales (Figure 2B, Table S7). There was no significant difference (t-test in R, p-value >0.32) in the content of Streptomycetales between two different extractions from the bone (Figure S2A, Table S8). Nor was there any difference in the abundance of Streptomycetales between subsets of short (<150 bp) and long (>150 bp) sequence reads (Figure S2B).

#### The cave sediment sample

We hypothesized that the bacterial sequences in the Neanderthal dataset might be derived from bacteria in the cave sediments. To test this hypothesis, we extracted bacterial DNA from cave sediment samples collected from the same place and G-layer as the remains of the Neanderthal bones. We amplified the SSU rRNA genes using universal primers and sequenced the amplified products. The 278 rRNA gene sequences were aligned and classified according to their positions in a maximum likelihood phylogenetic tree that included a set of reference species. The results indicated a similarly low level of diversity in the soil as in the Neanderthal sample, with 83% of the rRNA gene sequences classified as Actinobacteria and 9% as Proteobacteria (Figure 2A, Table S9). To ensure a good representation of the actinobacterial DNA, we also performed PCR amplifications using actinobacterial specific primers. However, in contrast to the dominance of Streptomycetales in the Neanderthal DNA, only a single such rRNA gene sequence was tentatively identified. Instead, Pseudonocardiales (40%) and Propionibacteriales (31%) dominated the bacterial rRNA gene sequences in the cave sediment sample **(** Figure 2B, Table S10).

#### The mammoth sample

For comparison, we included a dataset from the mammoth mandible (GPID 16317), which after clustering consisted of 0.2 million reads amounting to 21.2 Mb of sequence data (Table S1). Actinobacteria (27%) and Proteobacteria (31%) were the most abundant phyla in the mammoth sample as well (Table S9), although the majority of the actinobacterial sequence reads were assigned to the Micrococcales (74%) (Table S10).

### Actinobacterial Phylogeny

We performed phylogenetic analyses to directly compare the relationship of the actinobacterial rRNA gene sequences identified in the bone with those in the cave sediment sample, and also included other publicly available actinobacterial sequences. For this analysis, we used the 3.95 Gb dataset in which duplicate reads had been removed by the clustar method. To circumvent problems caused by low resolution in the phylogeny due to the short fragments of the rRNA reads in the Neanderthal sample, we assembled the individual reads into longer consensus sequences using a two-step procedure. We first extracted the 3,875 SSU rRNA gene sequences tentatively assigned to Streptomycetales in the BLASTn search against the eSILVA database (Table S7). In a second step, we filtered the reads for high BLAST score and then assembled them. By filtering reads carrying SNPs characteristic of the Streptomycetales, we reduced the risk of assembling reads belonging to different phyla. Moreover, we manually inspected the assembly and removed reads of low quality and misassembled reads. The result was a single full-length contig (here called SSU_Streptomycetales C11) that contained 2,754 sequence-reads and covered most of the SSU rRNA gene.

**Figure 3:**
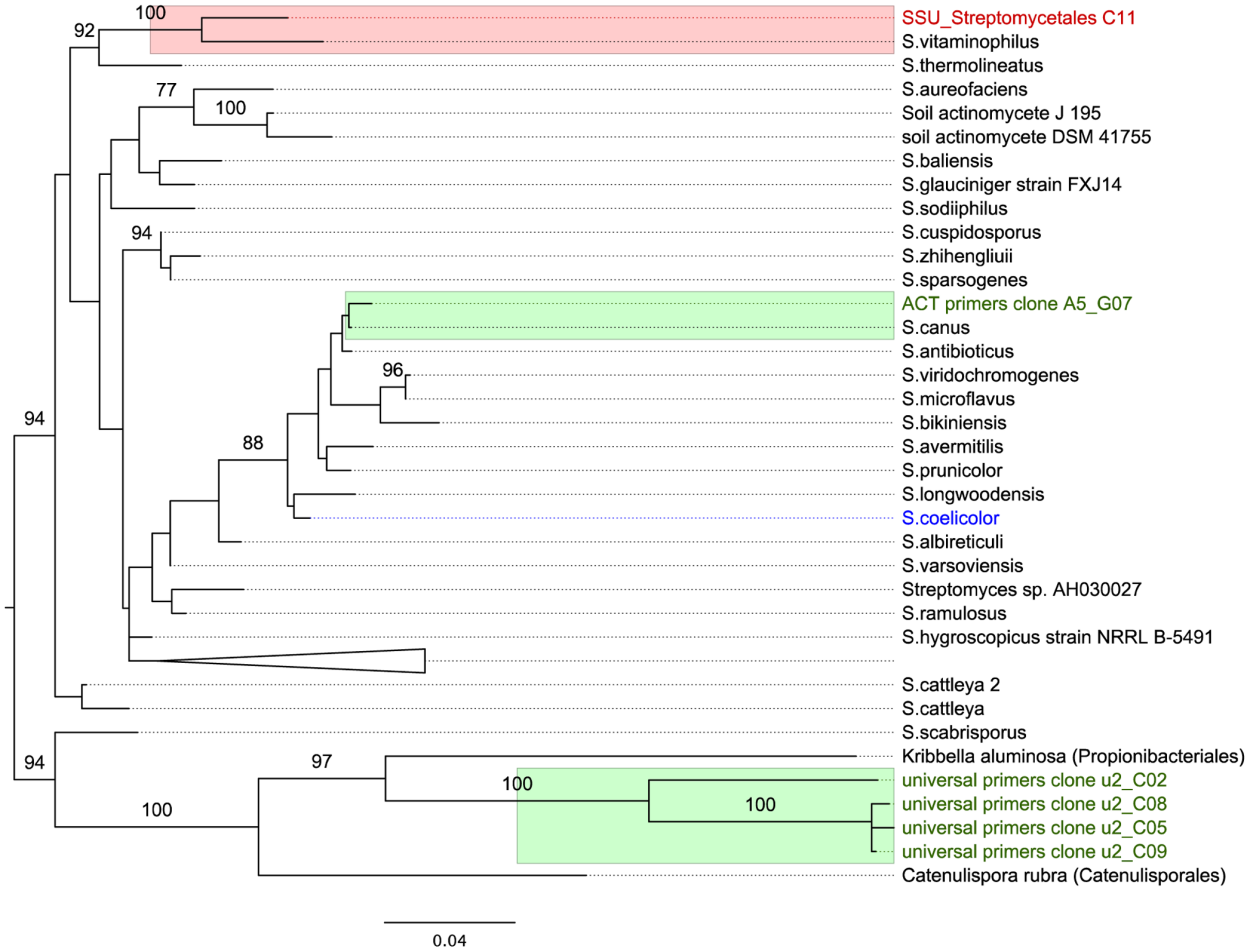
Phylogeny of rRNA gene sequences. The longest rRNA consensus sequence assembled from the Neanderthal data and assigned to Streptmycetales is shown in red (SSU_Streptomycetales C11). The rRNA gene sequences amplified from the cave sediment are shown in green colour. *Streptomyces coelicolor*, used as a reference in the alignment is shown in blue. The PCR-amplified sequences from the cave sediment are shown in green. “ACT primers clone A5_G07” refer to amplifications with the actinobacterial-specific primers, while the four sequences obtained from the universal primers 27f and 1492r are referred to as “universal primers clone u2_C02, 05, 08 and 09″. The phylogeny was inferred using the maximum likelihood method. Numbers refer to bootstrap support values higher than 75%.

The maximum likelihood phylogeny showed that the consensus C11 sequence clustered with *Streptomyces vitaminophilus* with 100% bootstrap support (Figure 3). The same placement was also observed for five shorter consensus sequences obtained if short and long reads were assembled separately (data not shown). The sequence identity of the consensus C11 sequence and *S. vitaminophilus* SSU rRNA gene was 97% over 1361 bp, and contained three indels of 13–15 bp. A comparison with the rRNA gene sequences of *S. griseus* and *S. coelicolor* indicated that the indels correspond to one insertion and two deletions in *S. vitaminophilus* (see positions 378, 644 and 1322 in Figure S3). Additionally, the consensus C11 and the *S. vitaminophilus* rRNA gene sequences differ by 29 substitutions, 1 bp indel and 9 homopolymer differences distributed across the genes (Figure S3). The read coverage of the consensus sequence was very high, with a mean of 261 and a minimum of 158 reads in the sequence aligned to *S. vitaminophilus*. The mean Phred quality score was 73, which corresponds to less than one error in 10,000 bp, and the minimum quality was 27 at one position. However, it should be recalled that these scores do not reflect the possibility of polymorphisms at any position, just the dominance of one particular base over all others.

The sole sequence assigned to *Streptomyces* in the cave sediment sample (ACT primers clone A5_G07) was placed distantly from the C11 consensus sequence in the phylogeny (Figure 3), with 24 substitutions and 2 homopolymer differences distributed across the aligned 645 bp (Figure S3). Using a similar procedure we also assembled sequence reads initially classified as *Pseudonocardia* or *Propionibacterium* in the taxonomy analyses into longer consensus sequences. Also in this case, the phylogenies confirmed the taxonomic assignments, but indicated that the Neanderthal consensus sequences clustered separately from the sediment rRNA gene sequences (Figures S4, S5).

### Substitution Patterns

A key question is whether the actinobacterial DNA is of ancient or more recent origin. We reasoned that it should be possible to use signs of DNA damage as a proxy for age. Previous studies have shown that C−>T changes are drastically elevated in the Neanderthal DNA compared to the substitution pattern in the modern human DNA due to increased frequencies of deamination of cytosine to uracil [7], [8]. Moreover, these studies showed that the rate of deamination is enhanced as much as 50- to 60-fold at the ends of the sequence reads, presumably because cytosine residues at the ends of molecules are more susceptible to deamination than cytosine residues inside the molecule [7].

**Figure 4:**
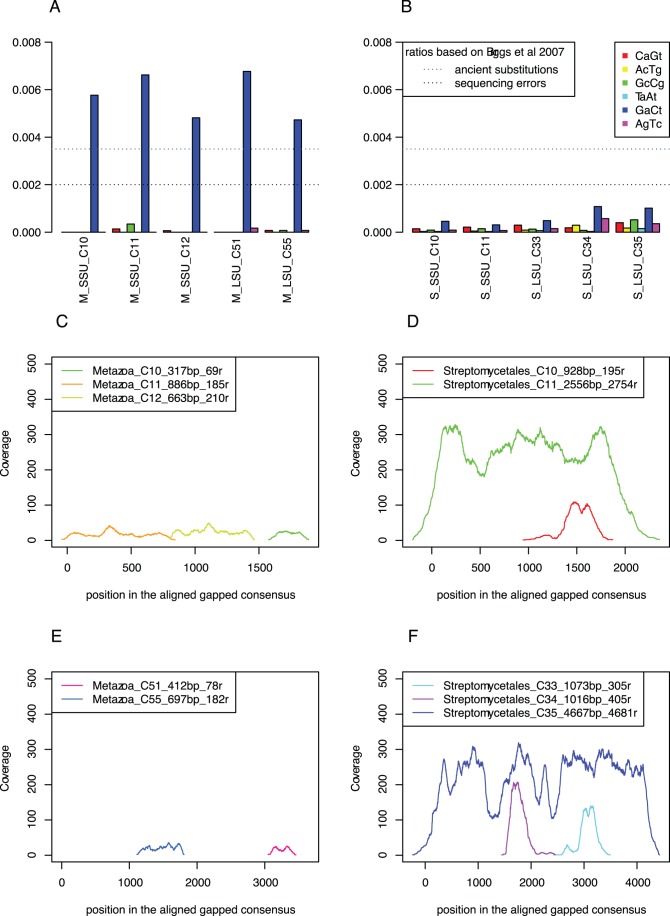
Substitution patterns in the Neanderthal and *Streptomyces* rRNA genes. Substitution frequencies inferred from the (A) Neanderthal and (B) Streptomyces small and large subunit rRNA gene contigs. Complementary substitutions ratios are reported together giving six groups in total. Vertical bars indicate the estimated level of sequencing errors. Coverage of sequence reads for the (C, D) small subunit and (E, F) large subunit rRNA gene sequences from (C, E) Metazoa and (D, F) Streptomycetales.

To infer substitution patterns in the Neanderthal and bacterial sequence reads, we identified single nucleotide changes in the individual reads through comparisons with the assembled SSU and LSU rRNA gene consensus sequences from *Streptomyces* (Figure 4). For comparison, we used three consecutive Neanderthal consensus sequences that covered most of the SSU rRNA and two shorter LSU rRNA contigs. The coverage of the bacterial rRNA gene sequences was about 250-fold, which was one order of magnitude higher than the coverage of the Neanderthal rRNA gene sequences. Because of the high coverage and the high frequency of frameshift errors at homopolymer sites in 454 sequence data that leads to the introduction of gaps, the total length of the bacterial rRNA gene sequences in the assembly are longer than the actual gene lengths (Figure 4).

We observed dramatically increased frequencies of C->T and G->A substitutions in the Neanderthal rRNA gene sequence reads, with an estimated 1.5 to 1.6×10^−2^ substitutions per site. To avoid problems caused by the inclusion of partial adaptor sequences in the bacterial sequence reads, we counted only nucleotide differences following the first 5 consecutive bases that were identical to the consensus sequence, and excluded sequence reads that contained more than 5 substitutions. Using this more stringent procedure, the frequency of changes in the Neanderthal rRNA gene sequence reads decreased to 5–7×10^−3^ substitutions per site. However, the strong over-representation of C−>T and G−>A substitutions was still very striking (Figure 4A). All other nucleotide changes in the Neanderthal rRNA gene sequence reads were <1 × 10^−3^ changes per site, i.e. approaching the error rate of the 454 instrument, which was previously estimated to be 4×10^−4^ errors per site [7].

The estimated frequencies of nucleotide substitutions for the bacterial rRNA gene sequences were in all cases in the range of the estimated level of sequencing errors, with no indications of an enhanced rate of C−>T and G−>A changes (Figure 4B). This suggests that the *Streptomyces* sequence reads originate from undamaged bacterial DNA. At this low level of diversity, only 381 of the 2754 reads contained nucleotide changes that were not at the end of the reads, of which we excluded 21 reads that contained more than 5 changes. Even if all of these relatively few reads containing a nucleotide change would be derived from the same gene, the diversity of strains represented in the assembly would be less than 1%. We did not attempt to infer substitution patterns for the other bacteria in the sample, due to much fewer reads per consensus rRNA gene sequence and higher levels of polymorphisms.

### Actinobacterial Collagenases and Proteases

The fate of archaeological bones, referred to as bone diagenesis, depends on the environmental conditions and the extent of microbial activity [9], [10]. Bone is a composite structure made of inorganic and organic components and the main protein is collagen, a protein with an atypically high content of proline, hydroxyproline and glycine. Collagenase is a key enzyme involved in the breakdown of collagen, and such an activity has been identified in *Streptomyces parvulus* and *Streptomyces griseus* [11], [12], [13], [14]. We hypothesized that the *Streptomyces*-like sequences might have been derived from a bacterium involved in the degradation of the bone, and therefore that collagenase genes might be present in the dataset.

#### Identification of collagenases

To identify such sequences, we first extracted all protein sequences annotated as collagenases in the non-redundant database (search for bacterial and archaeal proteins with ‘Name’ collagenase) and used these as queries in BLASTn searches against the Neanderthal metagenome (E<10^−5^). Metagenomic sequence reads thus identified were assembled to create longer consensus sequences. The same low levels of polymorphisms as in the rRNA gene assemblies were observed, with the frequencies of nucleotide changes in the individual reads being in the range of the error level of the instrument (Figure S6). The protein sequences were then used to search the MEROPS database for homologous proteins to be included in subsequent phylogenetic analyses. MEROPS is a designated protease domain database that contains 248,584 entries, including collagenases. We reasoned that protein domain designations would be more comprehensive and accurate in the MEROPS database and we therefore used this database (rather than the non redundant database) to annotate the consensus sequences and identify related sequences for the phylogenies. The four longest consensus sequences covered full-length collagenase genes and showed their best hits (E<10^−160^) to the *Streptomyces lividans* or the *Streptosporangium roseum* microbial collagenase V, from the M09 family.

**Figure 5:**
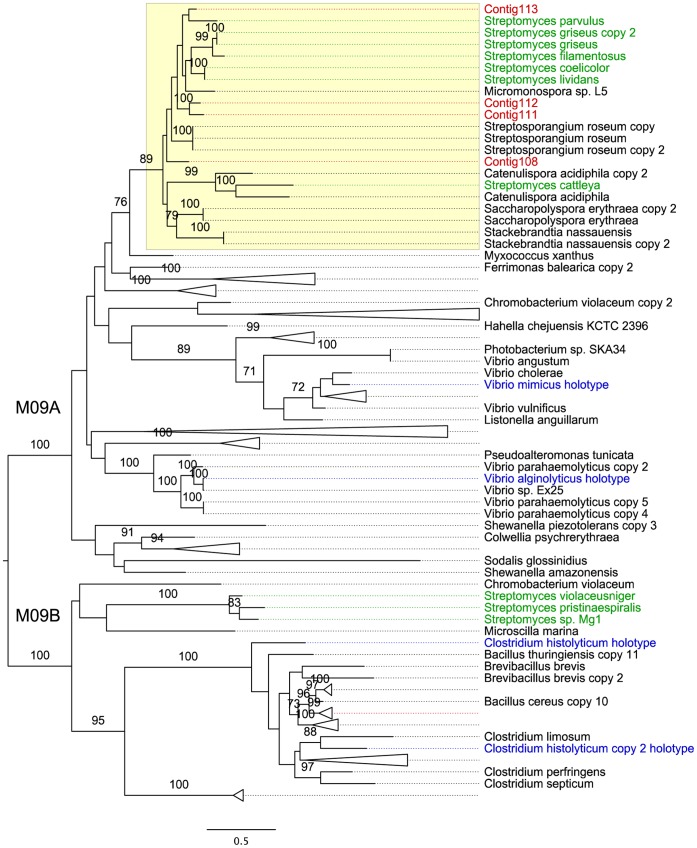
Phylogeny of microbial collagenases. Collagenase consensus sequences are coloured in red (Contigs 108, 111–113). The actinobacterial clade is highlighted in yellow and subfamilies M09A and B are indicated. The *Streptomyces* reference sequences from the MEROPS M09 family are shown in green and the family holotypes in blue. MEROPS references displayed with species names and (arbitrary) collagenase copy number. The phylogeny was inferred using the maximum likelihood method. Numbers refer to bootstrap support values higher than 75%.

Phylogenetic analysis confirmed that the consensus sequences clustered with *Streptomyces* and *Streptosporangium* collagenases of the M09A type with a bootstrap support of 89% (Figure 5). The less abundant collagen-degrading protein families included the M43 protease and the U32 collagenase family, each of which were assembled from up to 25 reads, or were singletons. Finally, we observed that the assembled collagenase sequences of the U32 type, which is broadly distributed in bacteria and eukaryotes, clustered with collagenases from bacterial species such as *Pseudomonas*, Enterobacteriaceae, *Acinetobacter* and Burkholderiales (Figure S7).

#### Identification of proteases

Collagenase degrades the collagen molecule into peptides, which, if used for nutrition, have to be further degraded and transported into the cell. As such, collagenases represent a small subset of a much larger family of proteases. To identify other types of proteases in *Streptomyces* we searched the MEROPS database with BLASTx using the Neanderthal sequence reads as the query (E<10^−10^). Protein family S33, which contains prolyl aminopeptidases that degrade proline-rich protein substrates, was assigned to about 6% of all reads (Table S11). Other abundant protease families identified in this search included collagen- (M23) and protein-degrading enzymes (M38, S09, S08, M20, M24, C26), as well as transport proteins (C44). However, most of the hits were to unassigned peptidases or non-peptidase homologs.

**Figure 6:**
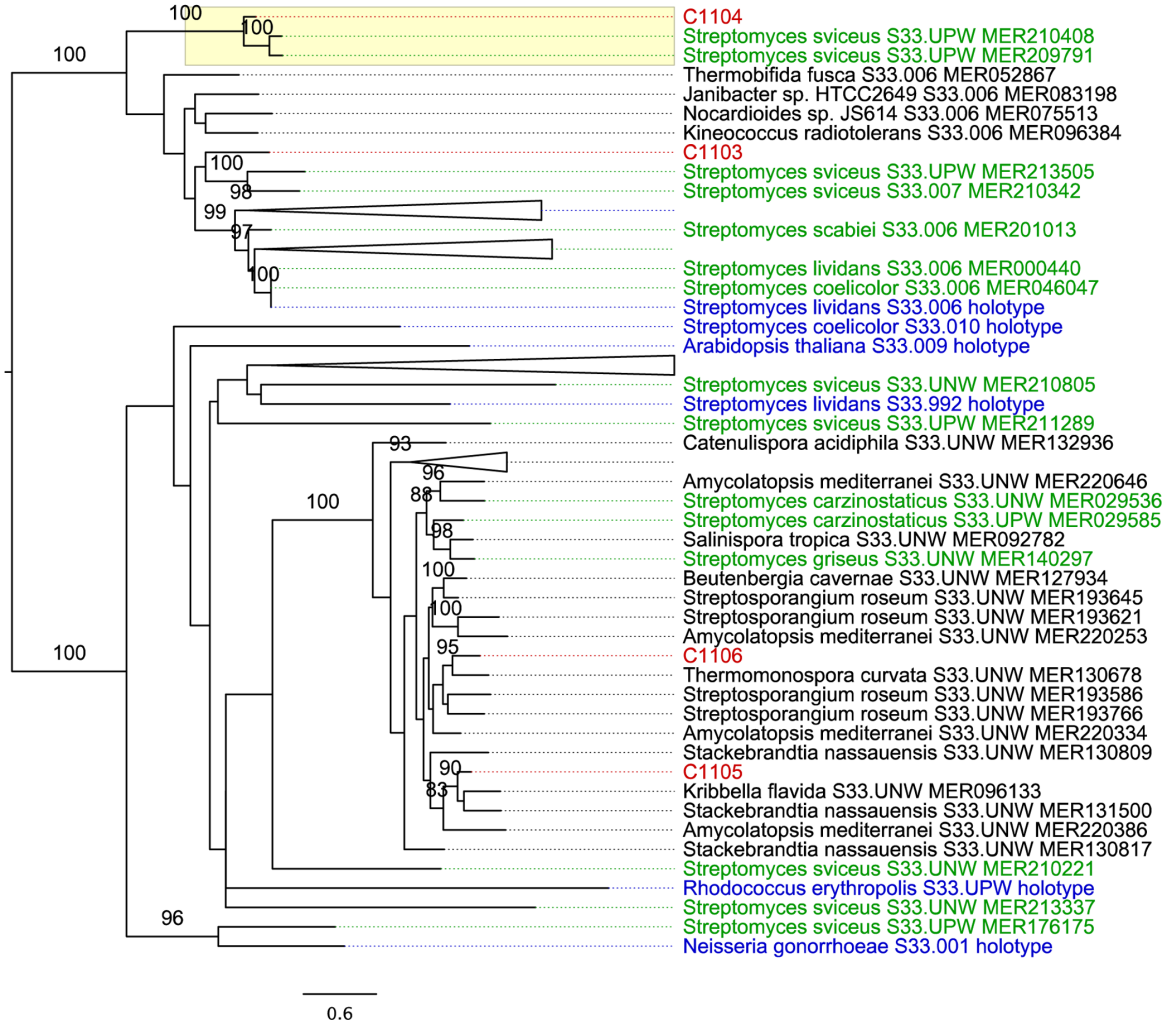
Phylogeny of prolyl aminopeptidases. Propyl aminopeptidase consensus sequences are coloured red (Contigs C1103–C1106). The clade containing contig C1104 and two *Streptomyces* species is shown in yellow. The *Streptomyces* reference sequences from MEROPS S33 family are shown in green and family holotypes in blue. MEROPS id is indicated for each sequence. The phylogeny was inferred using the maximum likelihood method. Numbers refer to bootstrap support values higher than 75%.

To investigate the taxonomic affiliations of the S33 protein family, the identified reads were assembled, yielding more than a thousand contigs in total. The consensus protein sequences of the four contigs that contained a majority of reads were compared to reference protein sequences of the S33 family in the MEROPs database in a maximum likelihood analysis (Figure 6). Two of the consensus sequences (C1103 and C1104) belonged to a clade with actinobacterial tripeptidyl peptidase B (S33.006) for which the holotype sequence is from *Streptomyces lividans* (100% bootstrap support). C1104 was placed as a sister taxa to *Streptomyces sviceus* with 100% bootstrap support and there was some support for a clustering also of C1103 with *Streptomyces*. To ensure the best possible match, we included all S33.006 sequences and all S33 sequences from *S. sviceus*, and top hits from the search against the MEROPs database where consensus sequences were used as queries.

The coverage of the consensus *Streptomyces*-like aminopeptidase and the collagenase sequences was about 25- and 40-fold, respectively, as compared to 200-fold coverage for the *Streptomyces*-like SSU and LSU rRNA consensus sequences (Table S12). Thus, the coverage levels of the *Streptomyces*-like sequences are roughly comparable if we assume one copy of each of the collagenase and aminopeptidase genes and six copies of the rRNA genes per genome [15], [16], [17], suggesting that these genes may be derived from the same *Streptomyces* species.

## Discussion

The recent large-scale genome projects on ancient organisms provide exciting opportunities to investigate and compare the microflora of archaeological bones of different ages and varied state of preservation. The findings presented in this study show that more than 90% of the identified rRNA gene sequences in the Neanderthal bone Vi33.16 were bacterial, with a majority being derived from Actinobacteria. Ancient DNA typically shows a different spectrum of nucleotide misincorporations than modern DNA, with atypically high rates of hydrolytic deaminations of C to T. These characteristics in nucleotide substitution patterns have helped distinguish the Neanderthal sequences from the human DNA, and it has also been observed in bacterial DNA from *Yersinia pestis* obtained from the teeth of victims of the Black Death during the 14^th^ century [18], [19].

To investigate the pattern of nucleotide misincorporations in the Neanderthal metagenome data, we compared individual rRNA sequence reads to the assembled consensus sequences. However, we soon realized that it was difficult to infer base changes at the ends of the sequence reads due to the presence of partial adaptor sequences in many reads. Single misassembled reads also had to be excluded not to inflate the frequency of base changes. To circumvent these problems we applied a more stringent procedure that only included base changes after the first 5 nucleotides that were identical in sequence to the consensus sequence. Although this approach reduced the overall level of changes, we observed the typical pattern of C to T changes and/or of its strand-equivalent substitution A to G in the Neanderthal DNA, consistent with chemical damage to the DNA after the death of the Neanderthal.

If the bacterial rRNA gene sequences were of the same age as the Neanderthal DNA, and had undergone similar DNA damage, a comparably high frequency of C to T changes was expected. This was not the case; the inferred substitution frequencies were extremely low for the *Streptomyces-*like sequence reads, in the range of the error level of the instrument. In viable cells, cytosine deamination errors are corrected by on-going DNA repair mechanisms, and it has been suggested that cells with low metabolic activity can persist over geological time scales [20]. This was thought to explain the lack of an increased frequency of C to T changes in DNA from Actinobacteria obtained from permafrost samples [20], [21], and could potentially also explain the lack of deamination errors in the bacterial fraction of the Neanderthal sample.

Ancient DNA is normally fragmented, and indeed the large majority of the Neanderthal sequence reads were of short sizes. The longer sequence reads were mostly of bacterial origin, but the short reads also contained bacteria in similar abundances. We considered the possibility that bacterial sequence reads of different sizes could be of different age. However, the same taxonomic composition and the same low substitution frequencies were observed for both short and long rRNA gene sequence reads, with no indications of increased rates of deamination damage in the short read pool. Based on this analysis, we conclude that the *Streptomyces*-like DNA was derived from live bacteria.

The inoculation and enrichment process in the bone could have occurred during the thousands of years when the fossil was kept in the cave sediment, or perhaps, during the past 20 years when the fossil has been located at a museum in Zagreb. Another possibility is that the identified bacterial DNA in the Neanderthal sample was derived from contaminations with cave sediments during the handling of the fossil. However, we have not detected a single case in which a bone contig sequence and a sediment clone sequence were placed as sister groups in the phylogenies, which rules out contamination with sediments as the source of the bacterial DNA. Rather, we hypothesize that *Streptomyces* was active early in the taphonomic process by utilizing bone as nutrient source, and then decreased its metabolic activity when conditions became unfavourable for growth. The identified *Streptomyces-*like collagenase sequences as well as many other proteases and peptidases, such as propyl aminopeptidase, are likely to have facilitated bacterial growth in the bone specimen. The bone collagen may first have been degraded into longer peptides by the collagenase and then further processed by propyl aminopeptidase and other proteases to shorter peptides.

Also of importance for long-term survival in the bone is the spore-forming ability of *Streptomyces* [22]. This is particularly relevant to this discussion since most of the *Streptomyces* population is thought to be present in the form of spores under natural conditions in the soil [23]. The presence of both viable and dormant forms of cells has also been reported from studies of deep subseafloor sediments [24], [25]. The type of spores that *Streptomyces* species produce lack some proteins and surface layers present in for example the *Bacillus*-type of spores and can thereby decompose more easily, releasing free DNA. Since the library preparation step of the Neanderthal DNA omitted the normal fragmentation step, the expectation is that free, fragmented DNA was preferentially sequenced [1]. Thus, some of the *Streptomyces* DNA may have been derived from spores that have lysed during the preparation of the DNA from the specimen [1].

Knowledge about the nucleotide composition of the microbiomes of archaeological samples is not only of scientific interest, but may also help implement strategies to eliminate contaminating bacterial DNA from ancient specimens. Even without such detailed knowledge, restriction digests targeted to GC-rich sequences had been applied to the Neanderthal DNA to reduce the bacterial DNA content and thereby increase the relative fraction of endogenous DNA [2]. In retrospect, we examined the relative fraction of bacterial rRNA gene sequences before and after the treatment with restriction enzymes and found these to be similar (Table S13). This may however not be surprising since rRNA genes are known not to be as biased in GC composition patterns as the rest of the genome due to secondary structural constraints and slow rates of sequence evolution, and will therefore be less affected than the majority of protein coding genes. Indeed, *in silico* digests with the restriction enzymes used in Mix2 and Mix1 of 11 *Streptomyces* genomes resulted in fragment lengths of only 14 bp and 30 bp on the average (Table S14). This suggests that most of the *Streptomyces* DNA was eliminated by these treatments.

Finally, our results differ from the first survey of the Neanderthal data in 2006 in that we estimated a much higher content of bacteria in the sample [1]. We reasoned that the difference in estimates might be due to the different methodologies used. The previous study [1] identified bacterial DNA by nucleotide BLASTn searches of individual reads against the public databases. Biased taxonomic representation of species in these databases, as well as nucleotide- instead of protein-level comparison hamper the detection of similarity. Moreover, short queries often fail to identify similar sequences with statistical significance, especially when used to search databases of large sizes, as confirmed by our *B. bovis* test dataset. Our approach differed in that we used designated rRNA gene sequence databases for the searches. The advantages are that rRNA genes are highly conserved in sequence, the databases contain orders of magnitude fewer sequences than the generalized sequence databases, yet have a much broader representation of taxa.

Clearly, much remains to be learned about the abundance, species richness and function of the microflora inhabiting human and animal fossils, and specifically what role the microbial community plays in the deterioration process. Here, there is much to hope from the initiatives worldwide to sequence ancient DNA from vertebrates. Studies of the microbial composition in archaeological samples are now feasible and should be of general interest to both microbial ecologists and archaeologists.

## Materials and Methods

### Data Sets and Samples

#### Ethics statement

All necessary permits were obtained for the described field studies. Bone as well as soil samples were collected under the auspices of the Memorandum of Understanding of December 8, 2006 between the Croatian Academy of Sciences and Arts, The Berlin Brandenburg Academy of Sciences and the Max Planck Institute for Evolutionary Anthropology on the determination and analysis of genome-wide DNA sequences in hominid fossils from Vindija, Croatia.

#### Sequence data

The Neanderthal sequence data set for fossil Vi33.16 (previously Vi-80) are available in the European Short Read Archive (EBI accession ERP002047). The two restriction enzyme treated 454 runs (Mix1 and Mix2) are available in the European Short Read Archive (EBI accession ERP000119, runs listed in table S6 in [2]). Mixture 1 was digested with *Bst*UI (CGCG), *Bsi*EI (CGRYCG) and *Hpy*99I (CGWCG) and Mixture 2 with *Bst*UI, *Bsi*EI plus *Taq*1 (TCGA), *Msp*I (CCGG), *Tau*I (GCSGC) and *Hin*P1I (GCGC). The mammoth dataset was downloaded from NCBI (GPID 16317, [3]).

#### Sediment samples

Sediment from Vindija cave, Croatia, layer G3 (the exact location of Neanderthal Vi-33.16) was sampled and stored in −80°C. DNA was extracted with an UltraClean soil DNA isolation kit (MoBio Laboratories, CA, USA). Approximately 0.5 g of soil was processed following the manufacturer’s protocol for maximum extraction yields.

### Data Processing

#### Adaptors

Oligonucleotide adaptors were retained in the Neanderthal dataset to designate the end of the template (see Figure 1 in [7]). Depending on the length of the template, they were fully present, partially present or missing from the sequence reads. Adaptors were removed from the Neanderthal sequence reads by alignment to a reference human sequence. Since the bacterial sequence reads lacked a reference genome, we removed adaptors by two procedures. For estimates of total bp, the end adaptor was mapped with PatMaN (v. 1.2 with default settings [26]), and trimmed from the read if it was situated at the end. For use in assemblies, a slower and more sensitive smalt indexing using shortest possible word length and step size was carried out (indexing run with the parameters – k 3– s 1; mapping run with default parameters; smalt version 0.5.7 http://www.sanger.ac.uk/resources/software/smalt/). Additionally, the substitution calculations were modified to ignore adaptor leftovers, as described below.

#### Quality

The quality of the Neanderthal reads was high (mean 27) and did not require any additional filtering steps. The quality of the many mammoth reads were low and the dataset was filtered to remove reads with mean quality below 20 using an in-house script, which increased the mean read quality from 19 to 25.

#### Clustering

To account for artificially multiplied reads, a known artifact of emulsion PCR [1], the sequence reads in the untreated Neanderthal data were first clustered using clustar, which was developed to suit this particular dataset. In short, the procedure consisted of a pairwise global alignment followed by single linkage clustering with a suitably selected cutoff (Table S2) for each emulsion set separately (Janet Kelso, personal communication). Additionally, a faster and less strict clustering with cd-hit version 3.1.2 using 97% identity threshold and 80% of the sequence in the alignment (cd-hit-454–c 0.97–aL 0.8, [27]), was applied to this and the three other datasets (Mix 1 and 2 and the mammoth dataset) for ease of comparisons of the different datasets. The stricter clustar clustering, which is probably more accurate, was only applied to the untreated Neanderthal dataset and used for the in-depth analysis of phylogeny, substitution patterns and proteases.

### Testing the Performance of BLAST

The *B. bovis* dataset consisted of 304,666 pyrosequencing reads from 1.6 Mb genome of *Bartonella bovis* (SRA accession SRR351456 [28], with mean read length of 106 bp. The genome was assembled using several kinds of sequencing data, with additional gap closure, producing high quality late draft that was estimated to contain 95% of the genome [28]. Completed bacterial and archaeal genomes were downloaded from NCBI (2211 genomes as of 8.03.2013) and formatted into a Blast database (P+). The shared sequence length of the assembled *B. bovis* genome and the *Bartonella* genomes in the databases were estimated by dividing the length of the shared blocks using progressive Mauve alignment [29] with the scaffold length. In order to investigate the impact of removing closely related sequences, a reduced database (P-) was prepared in which all *Bartonella* genomes were removed (*B. australis* Aust/NH1, *B. bacilliformis* KC583, *B. clarridgeiae* 73, *B. grahamii* as4aup, *B. henselae* Houston-1, *B. quintana* RM-11, *B. quintana* Toulouse, *B. tribocorum* CIP 105476 and *B. vinsonii berkhoffii* Winnie).

The performance of searches based on rRNA gene sequence similarities was evaluated by comparing the regular eSILVARef111 database (eSILVA+) to the same database without *Bartonella* sequences (eSILVA-). The assembler used 290,081 of the raw reads and their annotation was inferred from the position in the assembly. The expected number of rRNA hits was defined as all reads with at least 50 bp overlap with either the SSU (427 reads) or the LSU (837 reads) rRNA genes. False positives were defined as hits among the pool of reads after excluding the rRNA operons (287,772 reads). To make sure no adjacent sequence was left all reads that overlapped the operon boundaries plus 1000 bp flanking sequence were removed.

### Ribosomal RNA Sequence Analysis

#### PCR amplification, cloning and sequencing of the SSU rRNA gene

PCRs of SSU ribosomal rRNA gene sequences were conducted in a total reaction volume of 25 µl with two different sets of primers. Bacteria-specific primers 27f (5′-AGA GTT TGA TCC TGG CTC AG) and 1492r (5′-TAC GGY TAC CTT GTT ACG ACT T) were used at 0.5µM concentrations. Actinobacteria-specific primers ACT235f (5′-CGC GGC CTA TCA GCT TGT TG) and ACT878r (5′-CCG TAC TCC CCA GGC GGG G) were used at 0.5 µM concentrations. In both cases 200 µM concentrations of each deoxynucleoside triphosphate were used and approximately 10 ng of template was added. In the PCR with SSU rRNA gene primers 27f and 1492r, 4 µl of Phusion HF Buffer and 0.2 µl of Phusion Hot Start DNA polymerase (Finnzymes, Finland) were used, and the reaction was run starting with 30 s at 98°C, followed by 35 cycles of denaturation at 98°C for 5 s, annealing at 50°C for 5 s, and extension at 72°C for 60 s and the final extension at 72°C for 60 s. In the PCR with Actinobacteria-specific SSU gene primers ACT235f and ACT878r, 5 µl of Phire Reaction buffer and 0.5 µl of Phire Hot Start DNA polymerase (Finnzymes, Finland) were used, and reactions were run starting with 30 s at 98°C, followed by 35 cycles of denaturation at 98°C for 5 s, annealing at 60°C for 5 s, and extension at 72°C for 30 s and the final extension at 72°C for 60 s. Confirmation of the PCR product size was achieved by agarose gel electrophoresis of 1 µl of each PCR sample, staining with ethidium bromide, and visualization under UV light.

Products from the amplifications with universal primers were directly ligated into pCR-Blunt vectors using Zero Blunt PCR Cloning Kit (Invitrogen), and transformed into One Shot TOP10 Competent *Escherichia coli* cells (Invitrogen). Products from the actinobacterial primers were purified with Illustra GFX PCR DNA and Gel Band Purification Kit (GE Healthcare), and phosphorylated using the PCR Terminator End Repair Kit (Lucigen) following manufacturer’s recommendations for low amount of DNA. Products were then repurified, ligated into pSMART vectors using Clone Smart Blunt Cloning Kit (Lucigen) for 2 h, and transformed into XL2-Blue Ultracompetent *Escherichia coli* cells (Stratagene) using half the volume of competent cells (50 µl). DNA sequences were screened for vector and primer sequence and quality, and mate pairs were assembled using PhredPhrap’s default settings.

#### Identification and taxonomic assignment of the SSU rRNA gene sequence reads

To identify and assign rRNA gene sequences to a taxon, we performed BLASTn with an E-value threshold of 10^−10^ against a custom rRNA database based on SILVA Ref 111 [30] (EMBL release 111, March 2012). The LSURef111 and SSURef111NR databases in SILVA were modified prior to the searches by removing poorly aligned (align_quality_slv <75), potentially chimeric (pintail<100) sequences and unaligned ends (align_cutoff_head_slv, align_cutoff_tail_slv). This procedure is similar to the concept behind the truncated version of the SILVA database with the difference that a few sequences containing tRNA genes were deleted (see Table S15 for list of accession numbers of tRNA containing sequences). Our customized database excluded all sequences for which the taxonomic annotation differed at the domain level between the available taxonomies (Silva, EMBL, Greengenes, RDP), mostly due to different annotations of mitochondrial and chloroplast sequences. Taxonomically uninformative sequences, such as metagenome reads, were also excluded. To avoid pushing the classification closer to the root due to single misclassified sequences in the SILVA database, the taxonomic assignment of reads were based on an abundance criterion such that from all hits with the same best e-value, a lowest common ancestor was assigned based on the taxon associated with 50% or more of these hits. The few reads that had hits to different phyla were classified only to the level of the domain.

#### Sequence assembly

Reads identified by searches against the modified SILVARef111 database as described above were further filtered such that only reads that also had high BLAST scores (>75) were used for the assembly. The reads were binned according to their taxon identification, and separately for small and large rRNA subunit gene. After adaptor removal, assemblies were performed separately for each bin with PhredPhrap (-minmatch 30– maxmatch 55–max_subclone_size 50000–revise_greedy – vector_bound 20). Contigs used for the downstream analyses were manually inspected in Consed to assess potential misassemblies and levels of polymorphisms. The names designated for the resulting consensus sequences were chosen to provide information about the BLAST-based bin and the contig number within that bin. The Streptomycetales SSU rRNA gene sequences were also assembled using GS De Novo Assembler 2.3 (“cDNA” with default settings except for: “overlap identity” set to 99%, “reads limited to one contig” and “extending low depth overlaps”) 454 Life Sciences, Branford, CT [31]. This assembly generated a consensus sequence that was identical to the largest Streptomycetales SSU rRNA contig (C11), except for homopolymer errors.

#### Phylogenetic analysis

The rRNA consensus sequences and the rRNA gene sequences from the cave sediments were aligned with reference rRNA gene sequences using the integrated automatic aligner in ARB [32]. The alignments were analyzed using maximum likelihood methods implemented in RAxML [33] and the consensus sequences were classified according to their position in the resulting maximum likelihood tree.

Consensus SSU rRNA gene sequences longer than 500 bp and tentatively assigned to Pseudonocardiales, Propionibacterales, and Streptomycetales were added to the SILVA phylogenetic guide tree [30]. These alignments were manually refined using the closest neighbors as assigned by the most parsimonious placement of the sequence in the tree and exported. Alignment blocks were chosen after removal of poorly aligned regions such that regions that represented the PCR clones were retained (alignment files are available at the github repository, Table S16). Phylogenetic analyses were performed with maximum likelihood methods implemented in RAxML [33], using the GTRGAMMA model and with 100 bootstrap resampling replicates.

### Substitution Frequencies

The substitution patterns in the SSU and LSU rRNA gene sequences assigned to metazoan (Neanderthal) and bacteria (*Streptomyces*) were determined by comparing each of the individual reads to the consensus sequence. The ratio of the total number of changes to total bp in the contig was calculated for the 6 possible pairs of complementary changes, such as C−>T and A−>G. To account for unaligned reads due to partial adaptor sequences in the bacterial reads, the calculations included only nucleotide changes after the first 5 contiguous bases that agreed with the consensus and reads containing more than 5 changes in total were discarded.

### Analysis of Collagenases and Proteases

#### Identification of collagenase and protease sequences

Collagen sequence reads were identified in the Neanderthal metagenome with the use of a custom made dataset created by searching the NCBI protein database for collagenases (query ‘collagenase’ in field Name done on 28.09.2011) and extracting representative proteins from Bacteria and Archaea longer than 300 amino acids. After removal of duplicated copies from the same organism the database contained 106 sequences. To identify all proteases, the Neanderthal sequence reads were searched with BLASTx (e-value threshold 10^−10^) against the MEROPS peptidase protein sequence database (pepunit.lib downloaded on 09.03.2012) that consisted of 248,584 sequences. The results of the search were summarized by calculating number of hits assigned to each of the MEROP’s ids (for example S33.UNW).

#### Assembly

Individual sequence reads were grouped into subsets based on the identification as collagenases or proteases, separately for each family (for example S33). After adaptor removal, PhredPhrap was used to assemble (–minmatch 30 –maxmatch 55 –minscore 55 –max_subclone_size 50000 –revise_greedy –vector_bound 20) the reads identified for each type of protease into longer contigs. Assemblies used in the downstream phylogenetic analysis were manually inspected in Consed. Genes were called with the aid of prodigal [34] using metagenomic option for partial genes (–p meta).

#### Alignment and phylogeny

The consensus protein sequences were used to identify reference collagenase sequences in the MEROPS database (as of 6.12.2011) by an online BLAST with default settings [35]. All identified and inferred protein sequences were aligned using MAFFT (einsi, linsi and mafft-profile with default parameters, [36]). The alignments were manually checked to remove poorly aligned blocks and used for phylogenetic analysis with RAxML with 100 bootstrap replicates (−#100 -m PROTCATLG). The model for protein evolution was chosen using the script for model selection available from the RaxML website (http://www.exelixis-lab.org/).

### Scripts and Analysis Files

Lists of scripts and analysis files are listed in Table S16 and available in the github repository: https://github.com/kasiazar/NeandertalBoneMetagenome.

## Supporting Information

Figure S1 **Read length distribution of raw reads and identified ribosomal RNA reads.** Raw reads for mammoth and Neanderthal datasets, with separate y-axis scale on the right for the larger Neanderthal dataset (A). Vertical lines correspond to distribution peaks. Read length distribution of rRNA reads separated according to the presence/absence of the end-adaptor sequence (B). Reads classified as Neanderthal (Metazoa) plotted separately.
(PDF)

Figure S2 **Abundance variation of Streptomycetales reads.** The variation in abundance of SSU and LSU rRNA sequence reads classified as Streptomyces is shown for two different extractions from the bone, as detailed in Table S8 (A) and for two different subsets of sequence reads (B), with short reads defined as below 150 bp and long reads above 150 bp in size.
(PDF)

Figure S3 **Alignment of SSU rRNA gene sequences from** ***Streptomyces*** **.** Alignment of the consensus *Streptomyces* C11 sequence with reference SSU rRNA gene sequences from *S. vitaminophilus, S. griseus* and *S. coelicolor*, and sediment clones A5_G07 (actinobacterial primers) and u2_C02, 05, 08 and 09 (universal primers).
(PDF)

Figure S4 **Phylogeny of SSU rRNA gene sequences from Propionibacterineae.** The phylogeny includes previously sequenced SSU rRNA gene sequences as well as assembled consensus sequences assigned to Propionibacterineae, Pseudonocardineae and Actinomycetales. The consensus sequences are coloured red, with names including information about contig length, number of reads and whether the consensus sequence was assembled from the short read length pool with adaptor (A) or from the long read length pool with no adaptor (N). Sequences obtained by PCR amplification from the cave sediments are coloured in green. The phylogeny was inferred using the maximum likelihood method. Numbers refer to bootstrap support values higher than 75%.
(PDF)

Figure S5 **Phylogeny of SSU rRNA gene sequences from Pseudonocardineae.** The phylogeny includes previously sequenced SSU rRNA gene sequences as well as assembled consensus sequences assigned to Pseudonocardineae and Bacteria. The consensus sequences are coloured red, with names including information about contig length, number of reads and whether the consensus sequence was assembled from the short read length pool with adaptor (A) or from the long read length pool with no adaptor (N). Sequences obtained by PCR amplification from the cave sediments are coloured in green. The phylogeny was inferred using the maximum likelihood method. Numbers refer to bootstrap support values higher than 75%.
(PDF)

Figure S6 **Substitution patterns in the collagenases MEROPS M09 and proteases S33 genes.** Substitution frequencies inferred from the largest assembled contigs used for (A) collagenase phylogeny in Figure 5 and (B) protease phylogeny in Figure 6. Complementary substitutions ratios are reported together giving six groups in total. Vertical bars indicate the estimated level of sequencing errors. Coverage overview of the assembled gap-containing contigs aligned relative to the largest (C) collagenase Contig113 and (D) protease contig C1106.
(PDF)

Figure S7 **Phylogeny of collagenases belonging to the MEROPS family U32.** The phylogeny includes previously sequenced collagenase sequences as well as assembled consensus sequences. Colour coding refers to bone consensus sequences (red) and family holotypes from MEROPS (blue). The names of the MEROPS sequences include information about species, (arbitrary) collagenase copy number and a gene name for the *Escherichia coli* sequences. The contigs were numbered during assembly and the displayed name includes ORF parameters (# start # stop). The phylogeny was inferred using the maximum likelihood method. Numbers refer to bootstrap support values higher than 75%.
(PDF)

Table S1 Statistics for the raw and pre-processed datasets. The raw reads of the Mammoth dataset contains the 4 bp adaptor sequence at the beginning of each read. The raw reads of the Neanderthal dataset contains the 4 bp adaptor sequence at the beginning of each read and a complete 44 bp, a partial or no adaptor sequence at the end of each read.
(DOCX)

Table S2 Clustering with clustar for each of the library emulsions in the untreated Neanderthal dataset.
(DOCX)

Table S3 Performance tests of database searches. Performance of BLASTn searches using raw reads and reads in the assembly of the *Bartonella bovis* genome as queries against a database of more than 2,000 microbial genomes (P) and a cleaned up version of the SILVARef111 rRNA sequence database (eSILVA), respectively. Searches were performed against databases that included (P+ and eSILVA+) or excluded (P- and eSILVA-) related *Bartonella* sequences.
(DOCX)

Table S4 Precision and recall of searches against the eSILVA database. Reads in the assembly of the *Bartonella bovis* genome was used as queries with the e-value threshold set at e-10. The precision was calculated as TP/(TP+FP) and the recall as TP/(TP+FN). TP = True positives, calculated as the number of hits with > = 50 bp overlap with the SSU/LSU gene. FP = False positives, calculated as the number of reads showing hits that map outside the rRNA operons. FN = False negatives, calculated as true rRNA reads with no hits. SSU = Small Subunit rRNA; LSU = Large Subunit rRNA.
(DOCX)

Table S5 Classification of the identified small and large subunit rRNA gene sequences in the Neanderthal dataset at the domain-level. Included in the analyses were the untreated and restriction enzyme treated (Mix1 and Mix2) datasets.
(DOCX)

Table S6 Classification of the identified small and large subunit rRNA gene sequences in the Neanderthal dataset at the phylum-level. Included in the analyses were the untreated and restriction enzyme treated (Mix1 and Mix2) datasets. The category called “Bacteria” include phyla other than those specified, as well as reads that could not be classified below the domain level.
(DOCX)

Table S7 Classification of the identified small and large subunit rRNA gene sequences in the Neanderthal dataset within the Actinobacteria. Included in the analyses were the untreated and restriction enzyme treated (Mix1 and Mix2) datasets. The category called “Actinobacteria” include groups other than those specified, as well as reads that could not be classified below the phylum level.
(DOCX)

Table S8 List of the 454 library emulsions. Listed are the emulsions of two DNA extractions (extract and re-extract) and the sequencing runs corresponding to the extract (see table S2 for a list of sequencing runs for each emulsion set).
(DOCX)

Table S9 Classification of the identified rRNA gene sequences in the DNA extracted from the cave sediment sample and in the Mammoth dataset at the phylum-level. Universal primers (27f, 1492r) were used for the PCR amplifications of the small subunit rRNA gene sequences from the cave sediment sample. The category called “Bacteria” include phyla other than those specified, as well as reads that could not be classified below the domain level. The number of identified sequences is shown, with the percent given in parenthesis.
(DOCX)

Table S10 Classification of the identified rRNA gene sequences in the DNA extracted from the cave sediment and in the Mammoth dataset within the Actinobacteria. The number of identified sequences is shown, with the percent given in parenthesis.
(DOCX)

Table S11 The most abundant MEROPS protease families identified in the Neanderthal dataset. Family type peptidase refer to the protein that is the representative of the whole family. The ten most abundant MEROPS protease families were chosen from the list of MEROPS identities that had at least 0.5% abundance. The abundance was calculated from the number of hits to each of the families in a BLASTx search of all reads against the MEROPS peptidase protein sequence database (e-value threshold 10^−10^). The biological function description was taken directly from the MEROPS website.
(DOCX)

Table S12 Statistics for the assembled contigs of the rRNA, collagenase and aminopeptidase genes putatively assigned to Streptomyces. The relative gene copy numbers are based on the assumption that that the collagenase and aminopeptidase are singly-copy genes, while the rRNA genes are present in six copies per genome.
(DOCX)

Table S13 Comparison of the fraction of rRNA and total bacterial gene sequences in the Neanderthal datasets. The threshold for the identification of the rRNA genes was set to an e-value of <e-10 and a score of >100. The dataset in the upper row was clustered with cluster, while the three other datasets were clustered with cd-hit-454.
(DOCX)

Table S14 Mean lengths of genomic fragments after DNA digestions with restriction enzymes *in silico*. The restriction sites were mapped to the genomes with patman. Several *Streptomyces* genomes as well as a few other genomes of lower GC was included in the analysis.
(DOCX)

Table S15 List of sequences containing rRNAs in the truncated SILVA database, as detected by tRNAscan-SE.
(DOCX)

Table S16 List of scripts and analysis files available in the github repository. https://github.com/kasiazar/NeandertalBoneMetagenome.
(DOCX)

## References

[1] Green RE, Krause J, Ptak SE, Briggs AW, Ronan MT, et al (2006) Analysis of one million base pairs of Neanderthal DNA. Nature 444: 330–336. 17108958 10.1038/nature05336

[2] Green RE, Krause J, Briggs AW, Maricic T, Stenzel U, et al (2010) A draft sequence of the Neanderthal genome. Science 328: 710–722. 20448178 10.1126/science.1188021 PMC5100745

[3] Poinar HN, Schwarz C, Qi J, Shapiro B, MacPhee RDE, et al (2006) Metagenomics to paleogenomics: Large-scale sequencing of mammoth DNA. Science 311: 392–394. 16368896 10.1126/science.1123360

[4] Stiller M, Green RE, Ronan M, Simons JF, Du L, et al (2006) Patterns of nucleotide misincorporations during enzymatic amplification and direct large-scale sequencing of ancient DNA. Proc Natl Acad Sci U S A 103: 13578–13584. 16938852 10.1073/pnas.0605327103 PMC1564221

[5] Miller W, Drautz DI, Ratan A, Pusey B, Qi J, et al (2008) Sequencing the nuclear genome of the extinct woolly mammoth. Nature 456: 387–390. 19020620 10.1038/nature07446

[6] Keller A, Graefen A, Ball M, Matzas M, Boisguerin V, et al (2012) New insights into the Tyrolean Iceman’s origin and phenotype as inferred by whole-genome sequencing. Nat Commun 3: 698. 22426219 10.1038/ncomms1701

[7] Briggs AW, Stenzel U, Johnson PL, Green RE, Kelso J, et al (2007) Patterns of damage in genomic DNA sequences from a Neanderthal. Proc Natl Acad Sci U S A 104: 14616–14621. 17715061 10.1073/pnas.0704665104 PMC1976210

[8] Sawyer S, Krause J, Guschanski K, Savolainen V, Paabo S (2012) Temporal Patterns of Nucleotide Misincorporations and DNA Fragmentation in Ancient DNA. PLoS One 7. 10.1371/journal.pone.0034131 PMC3316601 22479540

[9] Child AM (1995) Microbial taphonomy of archaeological bone. Studies in Conservation 40: 19–30.

[10] Child AM (1995) Towards an understanding of the microbial decomposition of archaeological bone in the burial environment. Journal of Archaeological Science 22: 165–174.

[11] Tsiperovich OS, Mishunin IF (1973) Hydrolysis of the collagen from calf hide by a crystalline protease of Streptomyces griseus. Ukrainskii Biokhimicheskii Zhurnal 45: 151–155. 4196875

[12] Karpenko HF, Kastrykina TE, Tsyperovych OS (1977) Age difference of bone collagen in hydrolysis by Streptomyces griseus protease. Ukrainskii Biokhimicheskii Zhurnal 49: 76–80. 411202

[13] Karpenko HF, Kastrykina TF, Tsyperovych OS (1977) Study of bone tissue insoluble collagen hydrolysis by Streptomyces griseus protease using the method of N-terminal analysis. Ukrainskii Biokhimicheskii Zhurnal 49: 80–84. 407689

[14] Mishunin IF, Tsiperovich OS, Kuznetsova IM (1975) Hydrolysis of insoluble collagen of bull bones by Streptomyces griseus crystalline protease. Ukrainskii Biokhimicheskii Zhurnal 47: 226–232. 1884

[15] Rastogi R, Wu M, Dasgupta I, Fox GE (2009) Visualization of ribosomal RNA operon copy number distribution. BMC Microbiol. 9: 208. 10.1186/1471-2180-9-208 PMC2761929 19781086

[16] Ikeda H, Ishikawa J, Hanamoto A, Shinose M, Kikuchi H, et al (2003) Complete genome sequence and comparative analysis of the industrial microorganism Streptomyces avermitilis. Nat Biotechnol. 21: 526–31. 10.1038/nbt820 12692562

[17] Bentley SD, Chater KF, Cerdeño-Tárraga AM, Challis GL, Thomson NR, et al (2002) Complete genome sequence of the model actinomycete Streptomyces coelicolor A3(2). Nature 417(6885): 141–7. 12000953 10.1038/417141a

[18] Schuenemann VJ, Bos K, DeWitte S, Schmedes S, Jamieson J, et al (2011) Targeted enrichment of ancient pathogens yielding the pPCP1 plasmid of Yersinia pestis from victims of the Black Death. Proc Natl Acad Sci U S A. 108: E746–52. 10.1073/pnas.1105107108 PMC3179067 21876176

[19] Bos KI, Schuenemann VJ, Golding GB, Burbano HA, Waglechner N, et al (2011) A draft genome of Yersinia pestis from victims of the Black Death. Nature. 478: 506–10. 10.1038/nature10549 PMC3690193 21993626

[20] Johnson SS, Hebsgaard MB, Christensen TR, Mastepanov M, Nielsen R, et al (2007) Ancient bacteria show evidence of DNA repair. Proc Natl Acad Sci U S A. 104: 14401–5. 10.1073/pnas.0706787104 PMC1958816 17728401

[21] Willerslev E, Hansen AJ, Ronn R, Brand TB, Barnes I, et al (2004) Long-term persistence of bacterial DNA. Current Biology 14: R9–R10. 14711425 10.1016/j.cub.2003.12.012

[22] Goodfellow M, Williams ST (1983) Ecology of Actinomycetes. Annual Review of Microbiology 37: 189–216. 10.1146/annurev.mi.37.100183.001201 6357051

[23] Ensign JC (1978) Formation, properties, and germination of Actinomycete spores. Annual Review of Microbiology 32: 185–219. 10.1146/annurev.mi.32.100178.001153 360964

[24] Morono Y, Terada T, Nishizawa M, Ito M, Hillion F, et al (2011) Carbon and nitrogen assimilation in deep subseafloor microbial cells. Proc Natl Acad Sci U S A 108: 18295–18300. 21987801 10.1073/pnas.1107763108 PMC3215001

[25] Lomstein BA, Langerhuus AT, D’Hondt S, Jorgensen BB, Spivack AJ (2012) Endospore abundance, microbial growth and necromass turnover in deep sub-seafloor sediment. Nature 484: 101–104. 22425999 10.1038/nature10905

[26] Pruefer K, Stenzel U, Dannemann M, Green RE, Lachmann M, et al (2008) PatMaN: rapid alignment of short sequences to large databases. Bioinformatics 24: 1530–1531. 18467344 10.1093/bioinformatics/btn223 PMC2718670

[27] Li W, Godzik A (2006) Cd-hit: a fast program for clustering and comparing large sets of protein or nucleotide sequences. Bioinformatics 22: 1658–1659. 16731699 10.1093/bioinformatics/btl158

[28] Guy L, Nystedt B, Toft C, Zaremba-Niedzwiedzka K, Berglund EC, et al (2013) A Gene Transfer Agent and a Dynamic Repertoire of Secretion Systems hold the Keys to the Explosive Radiation of the Emerging Pathogen Bartonella. PLOS Genetics 9(3): e1003393. 23555299 10.1371/journal.pgen.1003393 PMC3610622

[29] Darling AE, Mau B, Perna NT (2010) progressiveMauve: multiple genome alignment with gene gain, loss and rearrangement. PLoS One 5. 10.1371/journal.pone.0011147 PMC2892488 20593022

[30] Pruesse E, Quast C, Knittel K, Fuchs BM, Ludwig W, et al (2007) SILVA: a comprehensive online resource for quality checked and aligned ribosomal RNA sequence data compatible with ARB. Nucleic Acids Research 35: 7188–7196. 17947321 10.1093/nar/gkm864 PMC2175337

[31] Meyer F, Paarmann D, D’souza M, Olson R, Glass E, et al (2008) The metagenomics RAST server–a public resource for the automatic phylogenetic and functional analysis of metagenomes. BMC Bioinformatics 9: 386. 18803844 10.1186/1471-2105-9-386 PMC2563014

[32] Ludwig W, Strunk O, Westram R, Richter L, Meier H, et al (2004) ARB: a software environment for sequence data. Nucleic Acids Research 32: 1363–1371. 14985472 10.1093/nar/gkh293 PMC390282

[33] Stamatakis A (2006) RAxML-VI-HPC: Maximum likelihood-based phylogenetic analyses with thousands of taxa and mixed models. Bioinformatics 22: 2688–2690. 16928733 10.1093/bioinformatics/btl446

[34] Hyatt D, Chen G-L, LoCascio PF, Land ML, Larimer FW, et al.. (2010) Prodigal: prokaryotic gene recognition and translation initiation site identification. BMC Bioinformatics 11. 10.1186/1471-2105-11-119 PMC2848648 20211023

[35] Rawlings ND, Barrett AJ, Bateman A (2010) MEROPS: the peptidase database. Nucleic Acids Research 38: D227–D233. 19892822 10.1093/nar/gkp971 PMC2808883

[36] Katoh K, Toh H (2008) Recent developments in the MAFFT multiple sequence alignment program. Briefings in Bioinformatics 9: 286–298. 18372315 10.1093/bib/bbn013

