# Supplementary figures and images for: No Ancient DNA Damage in Actinobacteria from the Neanderthal Bone
Source: PLoS One. 2013 May 3;8(5):e62799. doi: 10.1371/journal.pone.0062799 (PMC3643900; doi:10.1371/journal.pone.0062799)

A

## Read length distribution

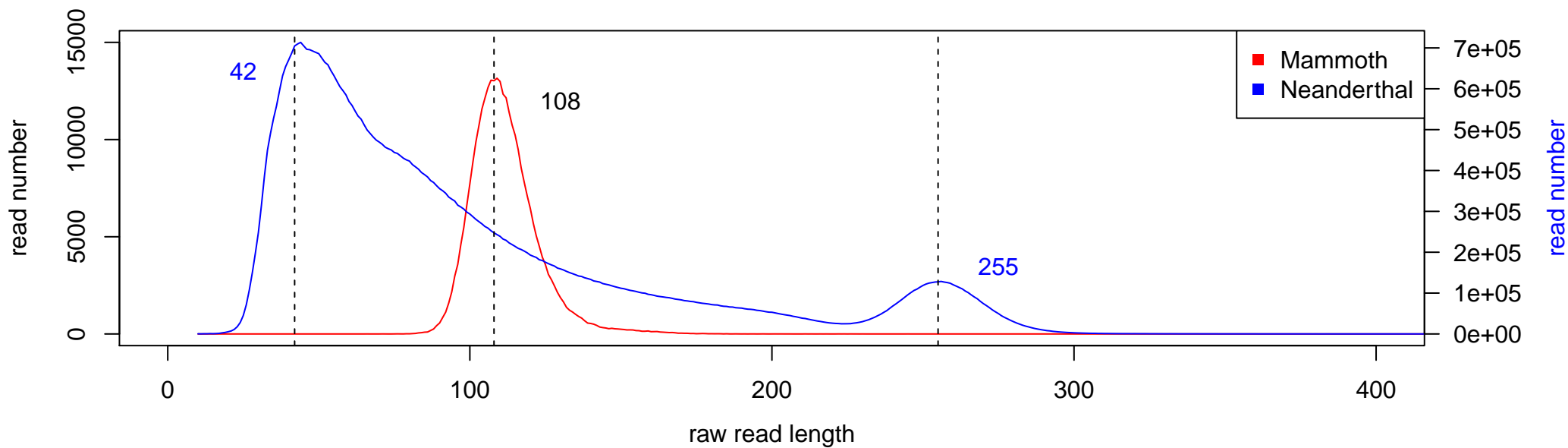

B

## rRNA read length distribution

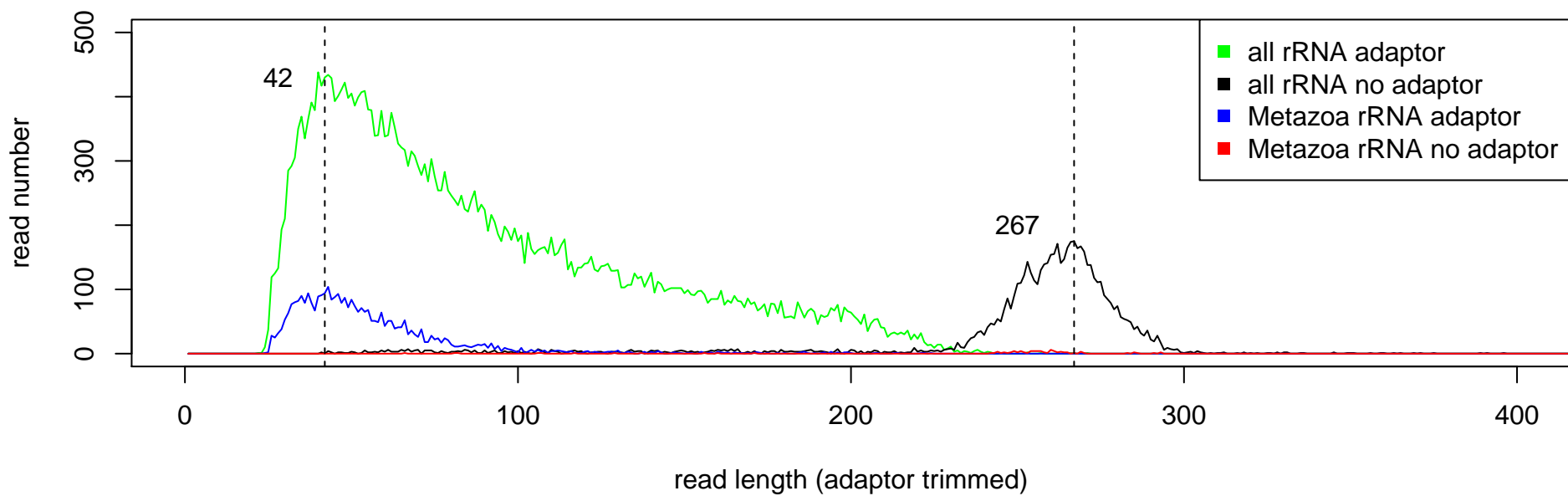

Supplement: Figure S1 — Read length distribution of raw reads and identified ribosomal RNA reads. Raw reads for mammoth and Neanderthal datasets, with separate y-axis scale on the right for the larger Neanderthal dataset (A). Vertical lines correspond to distribution peaks. Read length distribution of rRNA reads separated according to the presence/absence of the end-adaptor sequence (B). Reads classified as Neanderthal (Metazoa) plotted separately. (PDF) [file pone.0062799.s001.pdf]

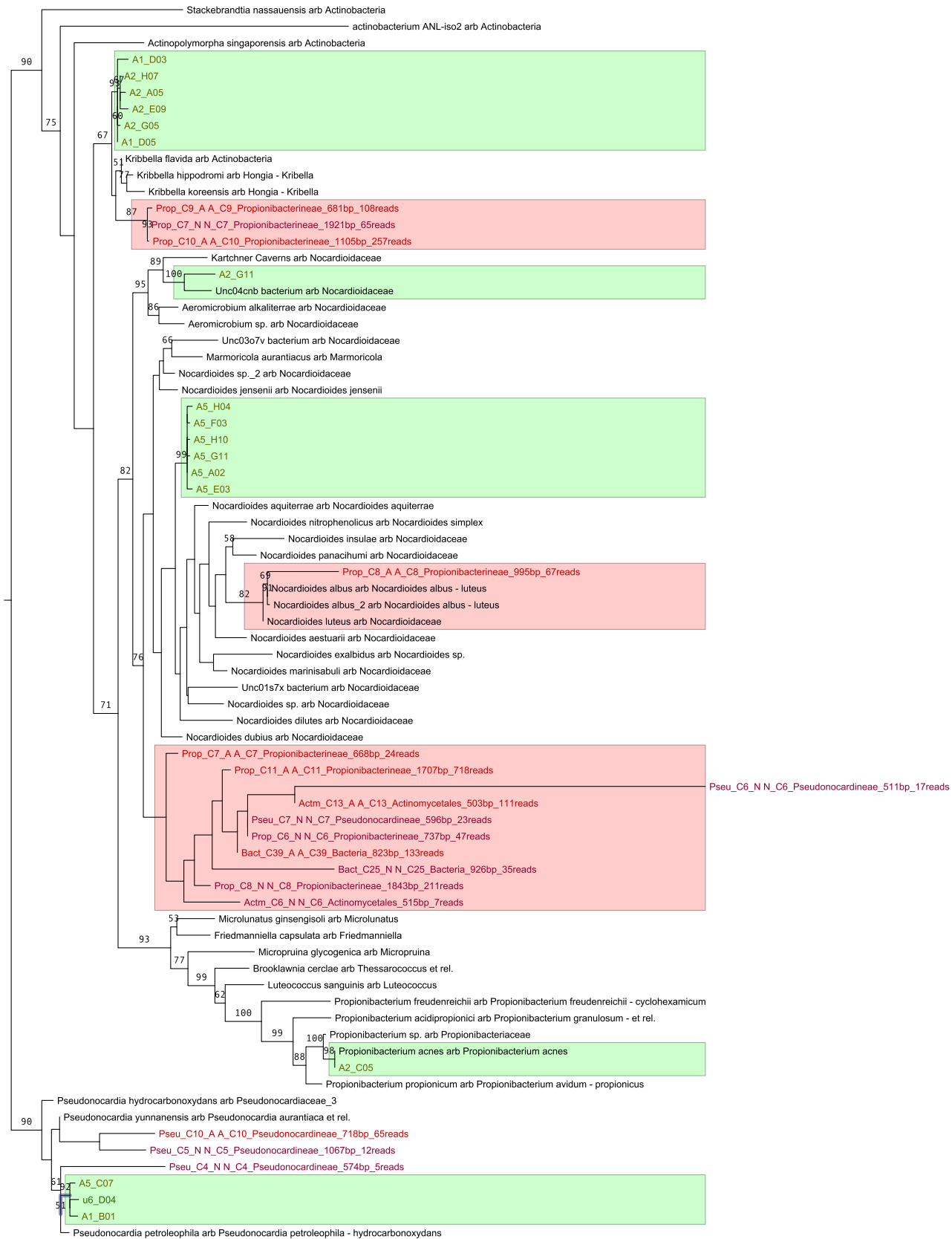

Supplement: Figure S4 — Phylogeny of SSU rRNA gene sequences from Propionibacterineae. The phylogeny includes previously sequenced SSU rRNA gene sequences as well as assembled consensus sequences assigned to Propionibacterineae, Pseudonocardineae and Actinomycetales. The consensus sequences are coloured red, with names including information about contig length, number of reads and whether the consensus sequence was assembled from the short read length pool with adaptor (A) or from the long read length pool with no adaptor (N). Sequences obtained by PCR amplification from the cave sediments are coloured in green. The phylogeny was inferred using the maximum likelihood method. Numbers refer to bootstrap support values higher than 75%. (PDF) [file pone.0062799.s004.pdf]

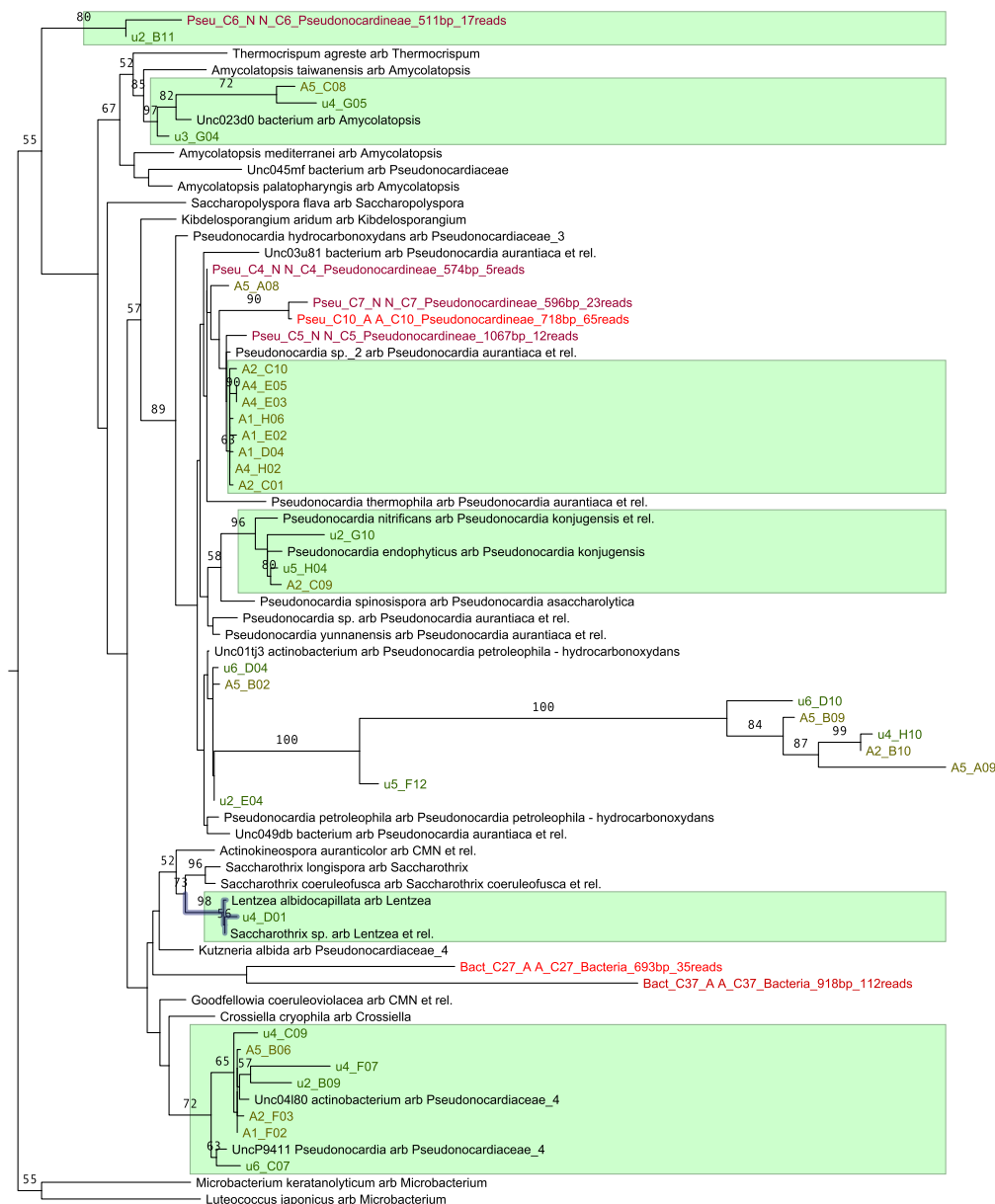

Supplement: Figure S5 — Phylogeny of SSU rRNA gene sequences from Pseudonocardineae. The phylogeny includes previously sequenced SSU rRNA gene sequences as well as assembled consensus sequences assigned to Pseudonocardineae and Bacteria. The consensus sequences are coloured red, with names including information about contig length, number of reads and whether the consensus sequence was assembled from the short read length pool with adaptor (A) or from the long read length pool with no adaptor (N). Sequences obtained by PCR amplification from the cave sediments are coloured in green. The phylogeny was inferred using the maximum likelihood method. Numbers refer to bootstrap support values higher than 75%. (PDF) [file pone.0062799.s005.pdf]

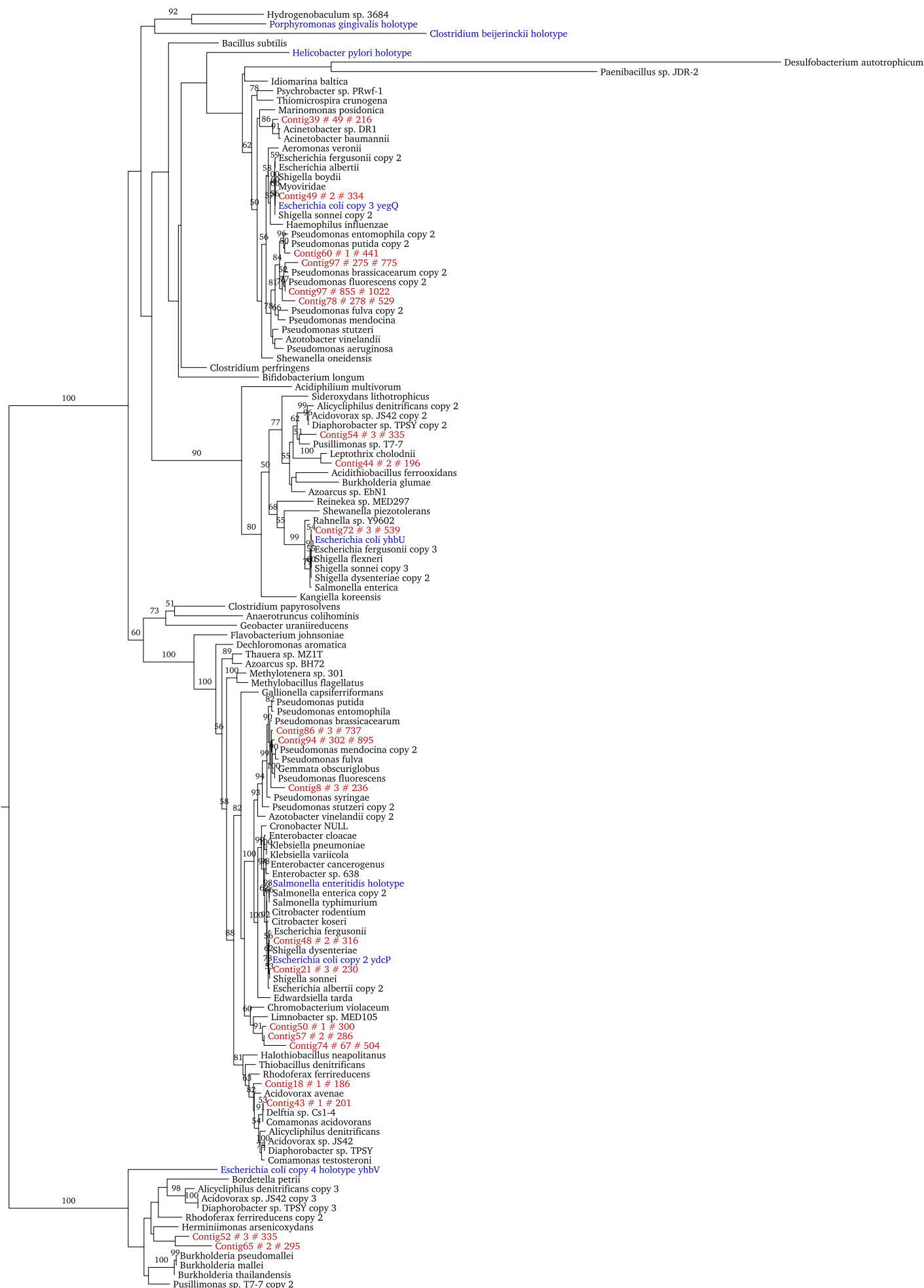

Supplement: Figure S6 — Substitution patterns in the collagenases MEROPS M09 and proteases S33 genes. Substitution frequencies inferred from the largest assembled contigs used for (A) collagenase phylogeny in Figure 5 and (B) protease phylogeny in Figure 6. Complementary substitutions ratios are reported together giving six groups in total. Vertical bars indicate the estimated level of sequencing errors. Coverage overview of the assembled gap-containing contigs aligned relative to the largest (C) collagenase Contig113 and (D) protease contig C1106. (PDF) [file pone.0062799.s006.pdf]

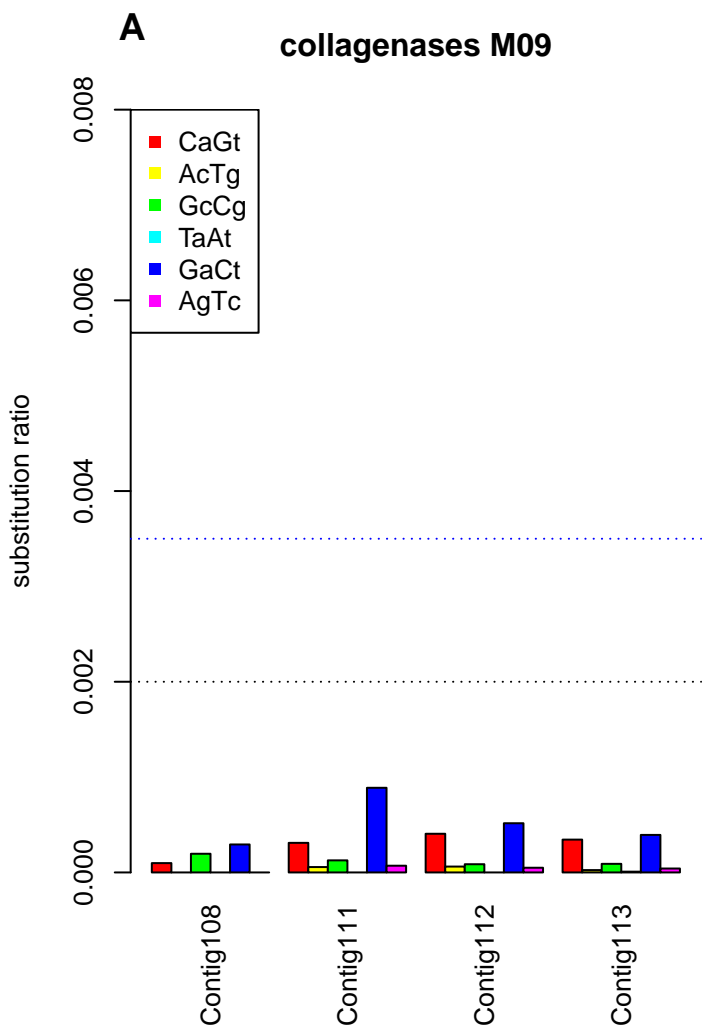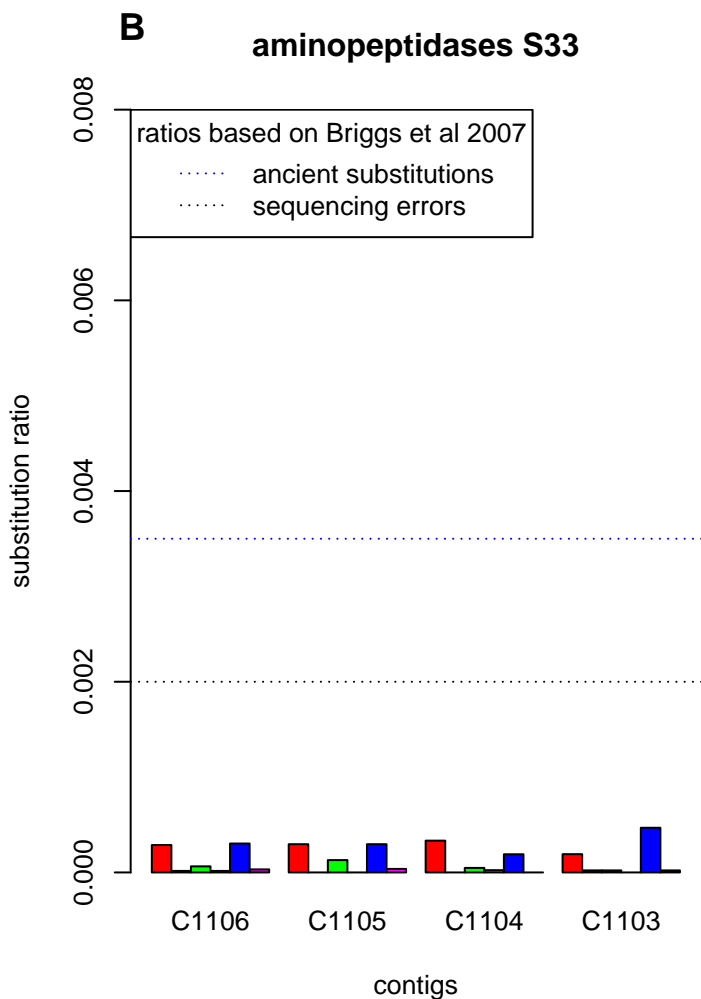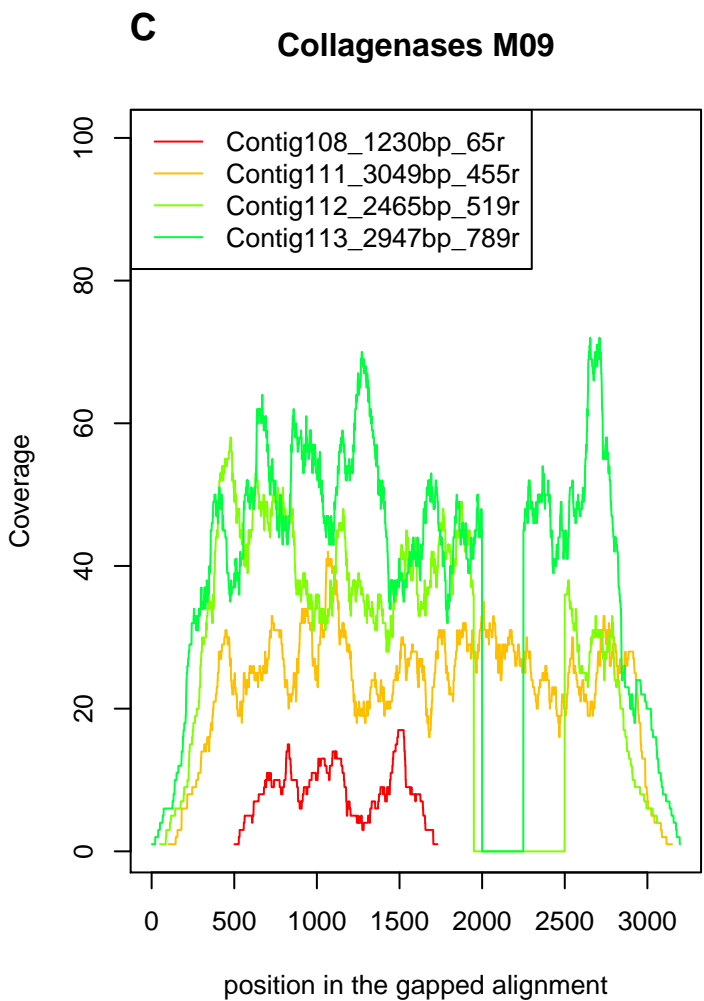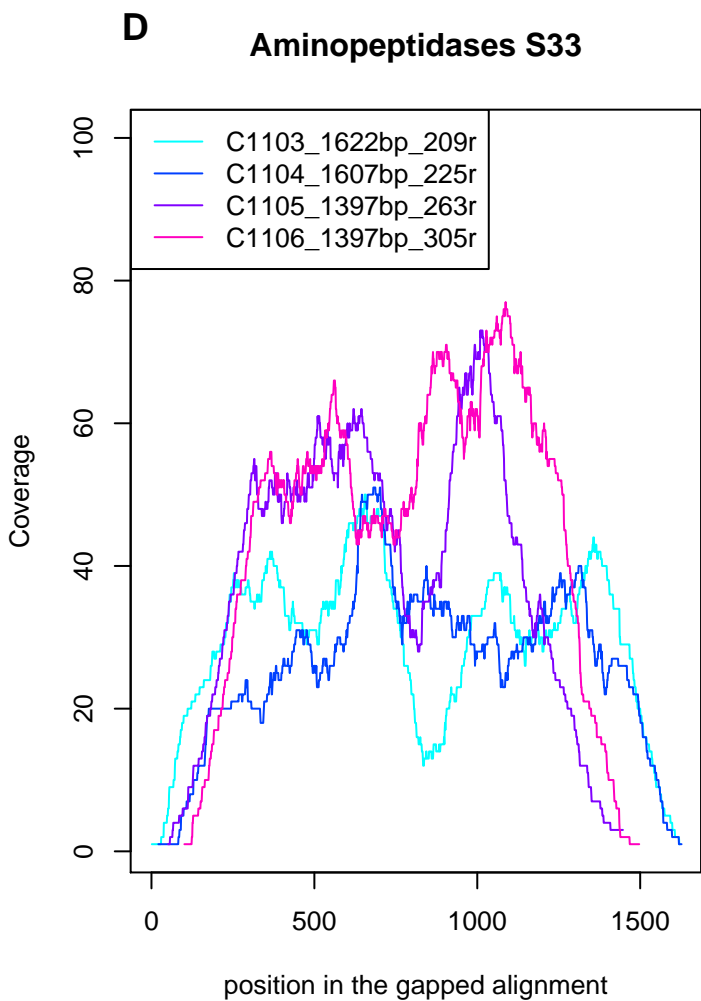

Supplement: Figure S7 — Phylogeny of collagenases belonging to the MEROPS family U32. The phylogeny includes previously sequenced collagenase sequences as well as assembled consensus sequences. Colour coding refers to bone consensus sequences (red) and family holotypes from MEROPS (blue). The names of the MEROPS sequences include information about species, (arbitrary) collagenase copy number and a gene name for the Escherichia coli sequences. The contigs were numbered during assembly and the displayed name includes ORF parameters (# start # stop). The phylogeny was inferred using the maximum likelihood method. Numbers refer to bootstrap support values higher than 75%. (PDF) [file pone.0062799.s007.pdf]
