# Supplementary material for: No Ancient DNA Damage in Actinobacteria from the Neanderthal Bone
Source: PLoS One. 2013 May 3;8(5):e62799. doi: 10.1371/journal.pone.0062799 (PMC3643900; doi:10.1371/journal.pone.0062799)
Supplement: Figure S2 — Abundance variation of Streptomycetales reads. The variation in abundance of SSU and LSU rRNA sequence reads classified as Streptomyces is shown for two different extractions from the bone, as detailed in Table S8 (A) and for two different subsets of sequence reads (B), with short reads defined as below 150 bp and long reads above 150 bp in size. (PDF) [file pone.0062799.s002.pdf]

A

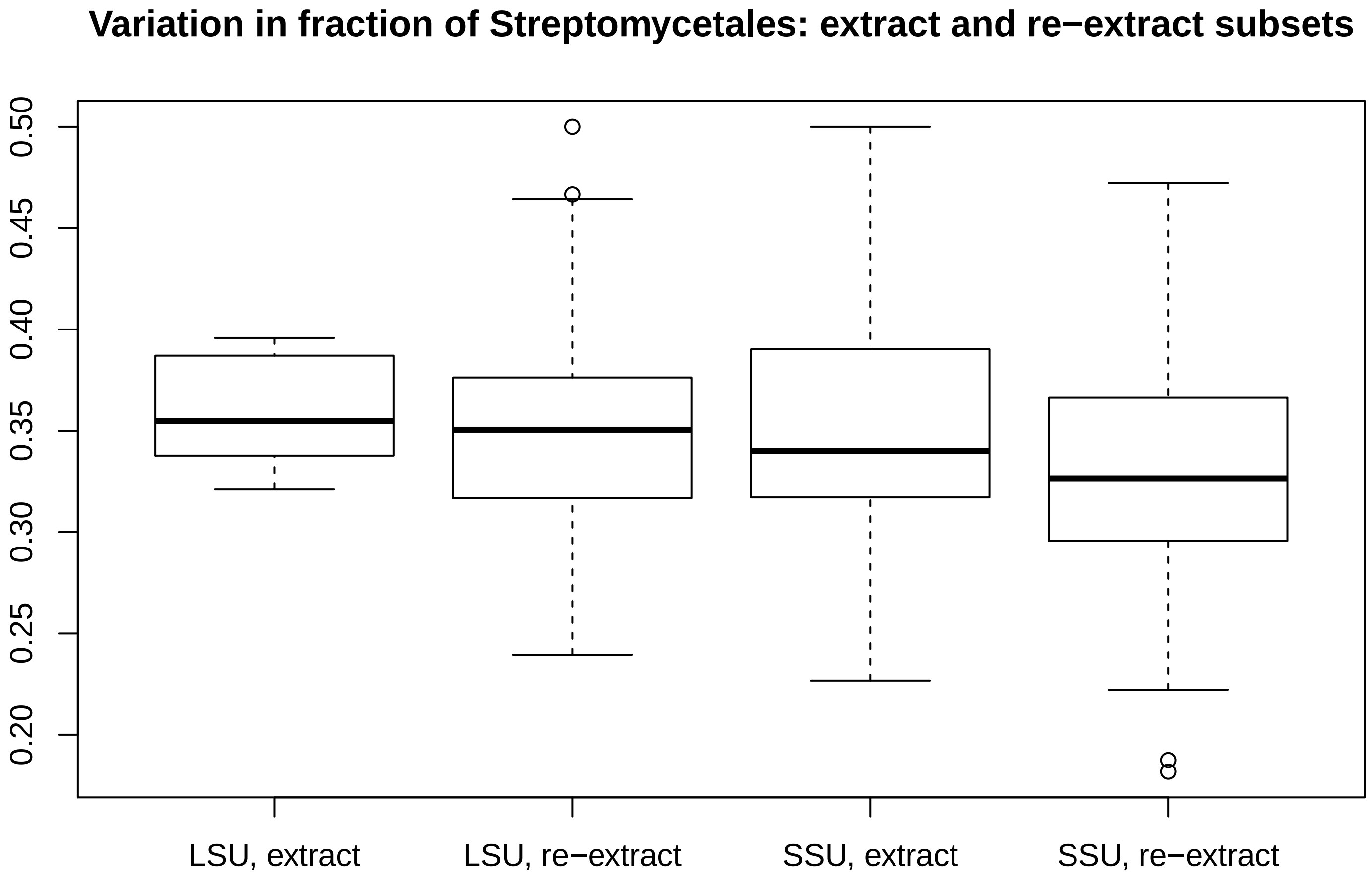
Variation in fraction of Streptomycetales: extract and re−extract subsets
0.20
0.25
0.30
0.35
0.40
0.45
0.50
LSU, extract
LSU, re−extract
SSU, extract
SSU, re−extract

B

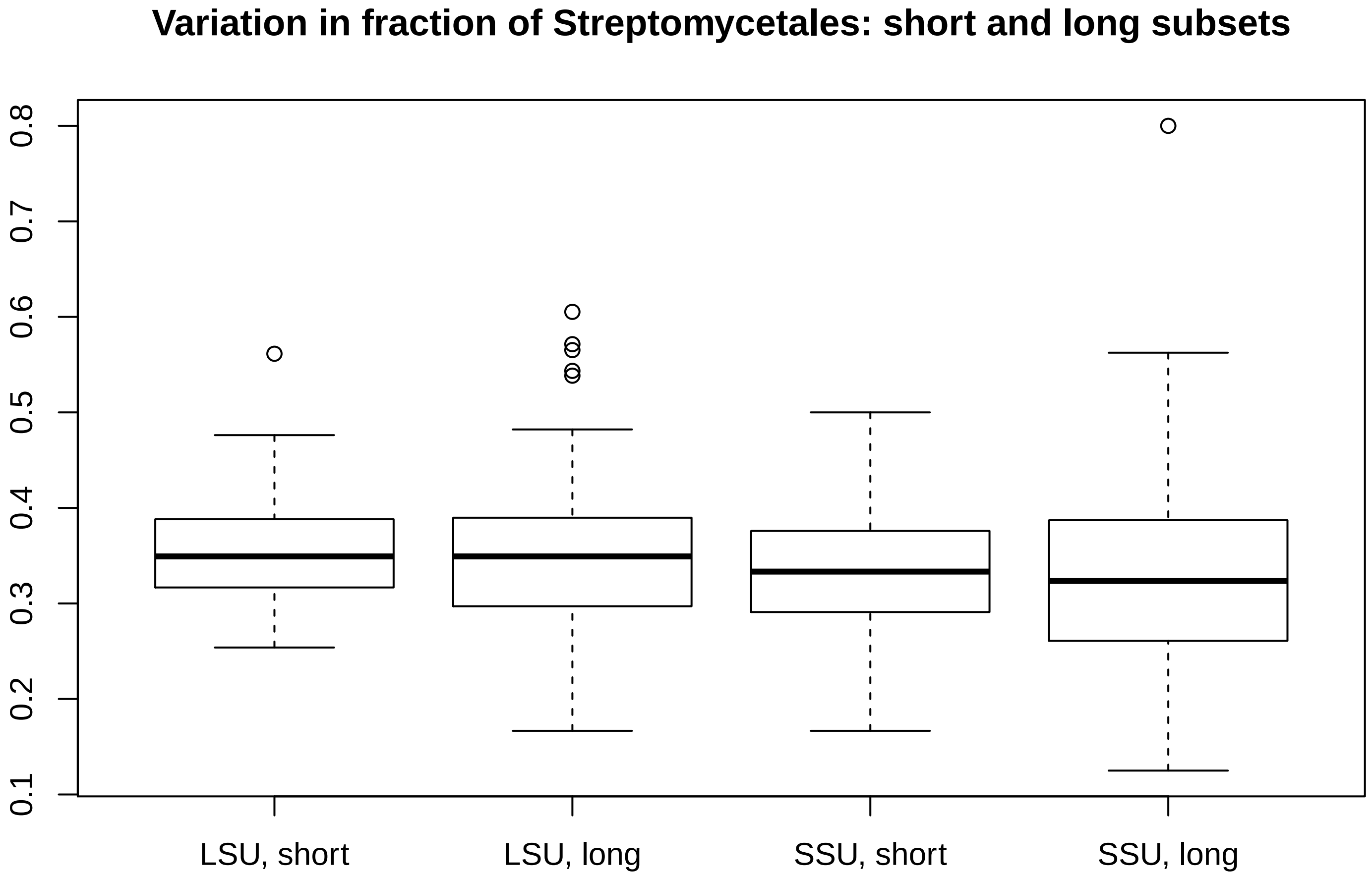
Variation in fraction of Streptomycetales: short and long subsets
0.1
0.2
0.3
0.4
0.5
0.6
0.7
0.8
LSU, short
LSU, long
SSU, short
SSU, long
