# Supplementary material for: No Ancient DNA Damage in Actinobacteria from the Neanderthal Bone
Source: PLoS One. 2013 May 3;8(5):e62799. doi: 10.1371/journal.pone.0062799 (PMC3643900; doi:10.1371/journal.pone.0062799)
Supplement: Figure S3 — Alignment of SSU rRNA gene sequences from Streptomyces . Alignment of the consensus Streptomyces C11 sequence with reference SSU rRNA gene sequences from S. vitaminophilus, S. griseus and S. coelicolor, and sediment clones A5_G07 (actinobacterial primers) and u2_C02, 05, 08 and 09 (universal primers). (PDF) [file pone.0062799.s003.pdf]

```
           1                            30
Scol        ------------------------------
Sgris       ------------------------------
SVit        ------------------------------
Strept_C11  GAACTCAACAGCGTGCCAAAAATCAACGCC
A5_G07      ------------------------------
u2_C05      ------------------------------
u2_C09      ------------------------------
u2_C02      ------------------------------
u2_C08      ------------------------------
```

```
           31                           60
Scol        ------------------------------
Sgris       ------------------------------
SVit        ------------------------------
Strept_C11  AGATTAGTTGATACCCCCGGACGGGGCCGC
A5_G07      ------------------------------
u2_C05      ------------------------------
u2_C09      ------------------------------
u2_C02      ------------------------------
u2_C08      ------------------------------
```

```
           61                           90
Scol        ------------------------------
Sgris       ------------------------------
SVit        ------------------------------
Strept_C11  GAATTTGTTTGTGGTCTTGTTTGGTGGTTC
A5_G07      ------------------------------
u2_C05      ------------------------------
u2_C09      ------------------------------
u2_C02      ------------------------------
u2_C08      ------------------------------
```

```
           91                          120
Scol        ------------------------------
Sgris       ------------------------------
SVit        ------------------------------
Strept_C11  CTTTGAAACACATATGCAAGGACGCTGGTG
A5_G07      ------------------------------
u2_C05      ------------------------------
u2_C09      ------------------------------
u2_C02      ------------------------------
u2_C08      ------------------------------
```

```
           121                         150
Scol        ------------------------------
Sgris       ------------------------------
SVit        ------------------------------
Strept_C11  GGGGCCCCGATTTTCCTCGGTGGCGGACCA
A5_G07      ------------------------------
u2_C05      ------------------------------
u2_C09      ------------------------------
u2_C02      ------------------------------
u2_C08      ------------------------------
```

```
           151                         180
Scol        ------------------------------
Sgris       ------------------------------
SVit        ------------------------------
Strept_C11  CCGCTCCTCCGCGTGTGTCATCCCTGATTA
A5_G07      ------------------------------
u2_C05      ------------------------------
u2_C09      ------------------------------
u2_C02      ------------------------------
u2_C08      ------------------------------
```

```
           181                         210
Scol        -----------UUCACGGAGAGUUUGAUCC
Sgris       -----------UUCACGGAGAGUUUGAUCC
SVit        ------------------------------
Strept_C11  CAGGGGAAGCATTCACGGAGAGTTTGATCC
A5_G07      ------------------------------
u2_C05      ------------------------------
u2_C09      ------------------------------
u2_C02      ------------------------------
u2_C08      ------------------------------
```

```
           211                         240
Scol        UGGCUCAGGACGAACGCUGGCGGCGUGCUU
Sgris       UGGCUCAGGACGAACGCUGGCGGCGUGCUU
SVit        ------------------------------
Strept_C11  TGGCTCAGGACGAATGCTGGCGGCGTGCTT
A5_G07      ------------------------------
u2_C05      ------------------------------
u2_C09      ------------------------------
u2_C02      ------------------------------
u2_C08      ------------------------------
```

```
           241                         270
Scol        AACACAUGCAAGUCGAACGAUGAACCACUU
Sgris       AACACAUGCAAGUCGAACGAUGAAGCCUUU
SVit        ------------------------------
Strept_C11  AACACATGCAAGTCGAACGGTGAAGCCTTT
A5_G07      ------------------------------
u2_C05      ------------------------------
u2_C09      ------------------------------
u2_C02      ------------------------------
u2_C08      ------------------------------
```

```
           271                         300
Scol        CGGUGGGGAUUAGUGGCGAACGGGUGAGUA
Sgris       CGGGGUGGAUUAGUGGCGAACGGGUGAGUA
SVit        ------GGATCAGTGGCGAACGGGTGAGTA
Strept_C11  CGGGGTGGATCAGTGGCGAACGGGTGAGTA
A5_G07      ------------------------------
u2_C05      ------GGATGAGTGGCGAACGGGTGAGTA
u2_C09      ------GGATGAGTGGCGAACGGGTGAGTA
u2_C02      ------ACTCGAGTGGCGAACGGGTGAGTA
u2_C08      ------GGATGAGTGGCGAACGGGTGGGTA
                  GGAT AGTGGCGAACGGGTGAGTA
```

```
           301                         330
Scol        ACACGUGGGCAAUCUG-CCCUUCACUCU-G
Sgris       ACACGUGGGCAAUCUG-CCCUUCACUCU-G
SVit        ACACGTGGGCAATCTG-CCTCGTACTTT-G
Strept_C11  ACACGTGGGCAATCTGCCCCCAGATTCTGG
A5_G07      ------------------------------
u2_C05      ACACGTGGGTAACCTG-TCCTGTACTTT-G
u2_C09      ACACGTGGGTAACCTG-TCCTGTACTTT-G
u2_C02      ACACGTGAGTAATCTG-CCTTTGGCTTT-G
u2_C08      ACACGTGGGTAACCTG-TCTTGTACTTT-G
            ACACGTGGG AA CTG CCC   ACTTT G
```

```
           331                         360
Scol        GGACAAGCCCUGGAAA-CGGGGUCUAAUAC
Sgris       GGACAAGCCCUGGAAA-CGGGGUCUAAUAC
SVit        GGATAACCCGGGGAAA-CTCGGGCTAATAC
Strept_C11  GGACAACCCGGGGAAACCTCGGGCTAATAC
A5_G07      ------------------------------
u2_C05      GGATAAACCCTGGAAA-CGGGGTCTAATAC
u2_C09      GGATAAGCCCTGGAAA-CGGGGTCTAATAC
u2_C02      GGATAAGCCTCGGAAA-CGGGGTCTAATAC
u2_C08      GGATAAGCCCTGGAAA-CGGGGTCTAATAC
            GGATAAGCCC GGAAA CGGGG CTAATAC
```

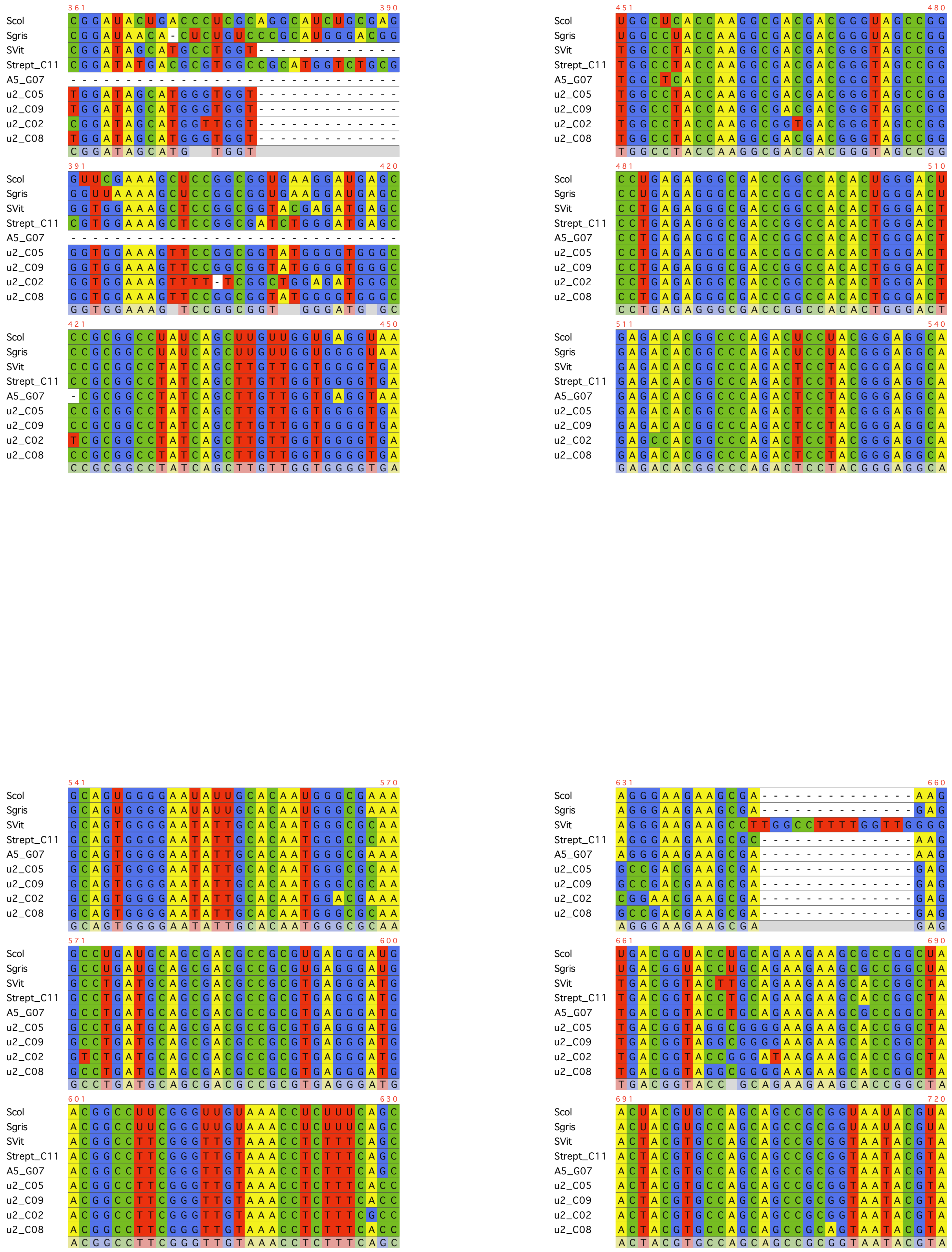

Scol
Sgris
SVit
Strept_C11
A5_G07
u2_C05
u2_C09
u2_C02
u2_C08
361 390
391 420
421 450
451 480
481 510
511 540
541 570
571 600
601 630
631 660
661 690
691 720

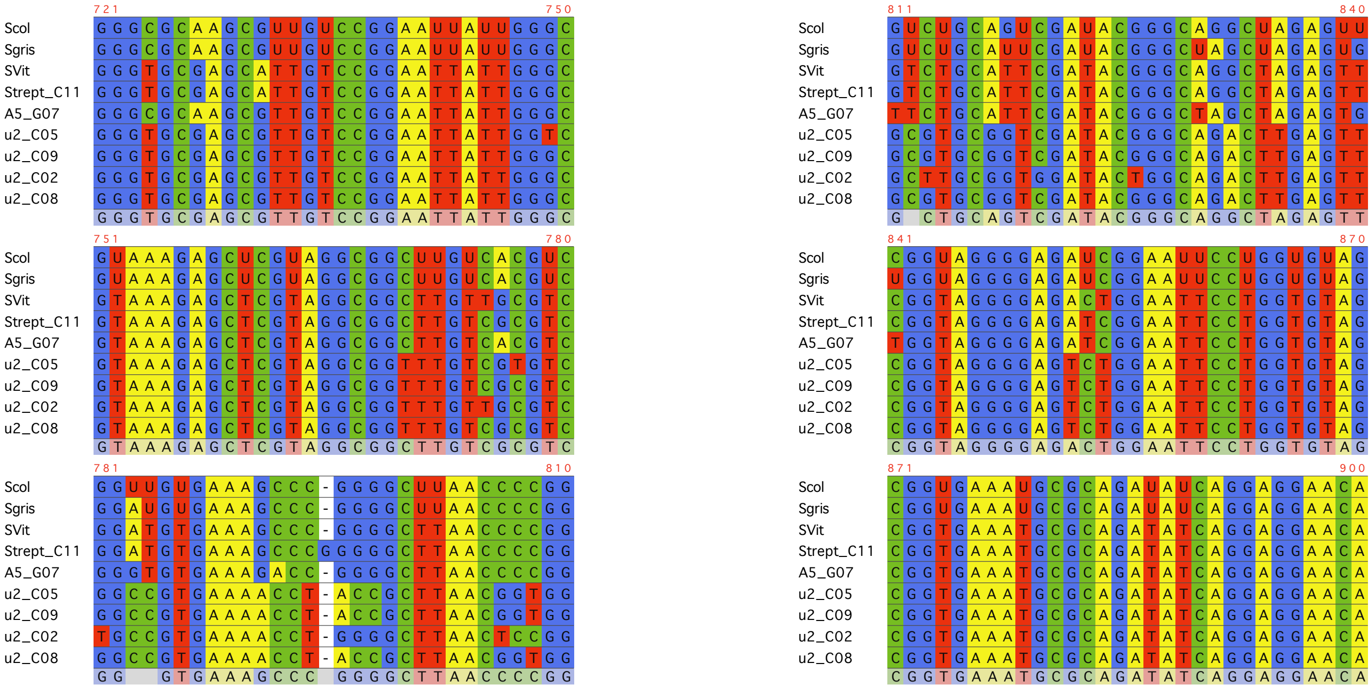

721
750
751
780
781
810
811
840
841
870
871
900
Scol
Sgris
SVit
Strept_C11
A5_G07
u2_C05
u2_C09
u2_C02
u2_C08

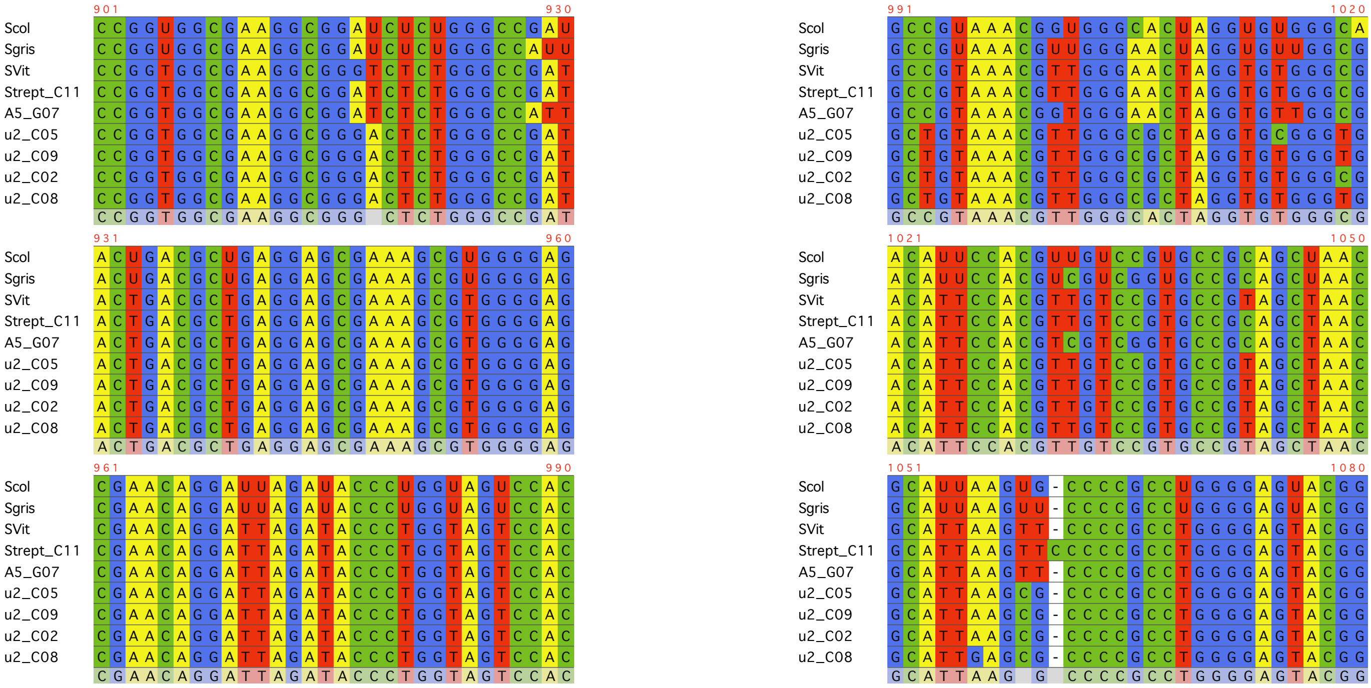

901
930
931
960
961
990
991
1020
1021
1050
1051
1080
Scol
Sgris
SVit
Strept_C11
A5_G07
u2_C05
u2_C09
u2_C02
u2_C08

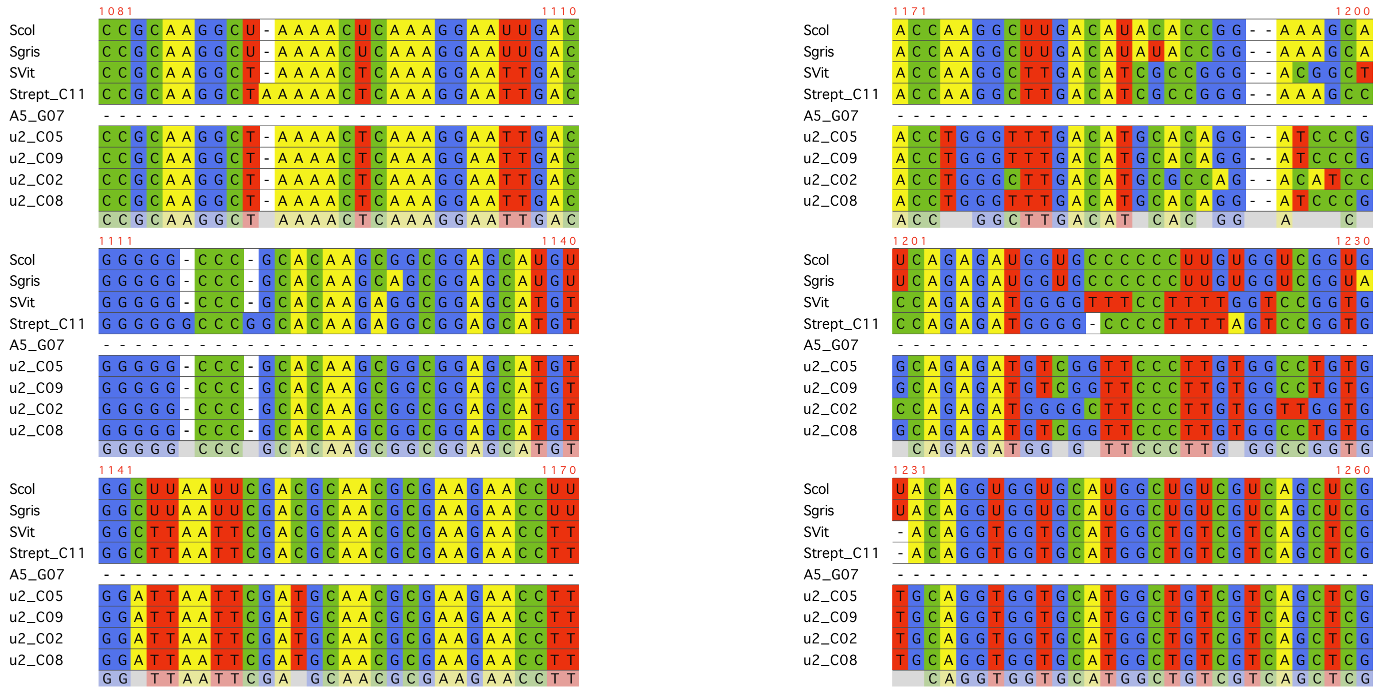
1081
1110
1111
1140
1141
1170
1171
1200
1201
1230
1231
1260
Scol
Sgris
SVit
Strept_C11
A5_G07
u2_C05
u2_C09
u2_C02
u2_C08

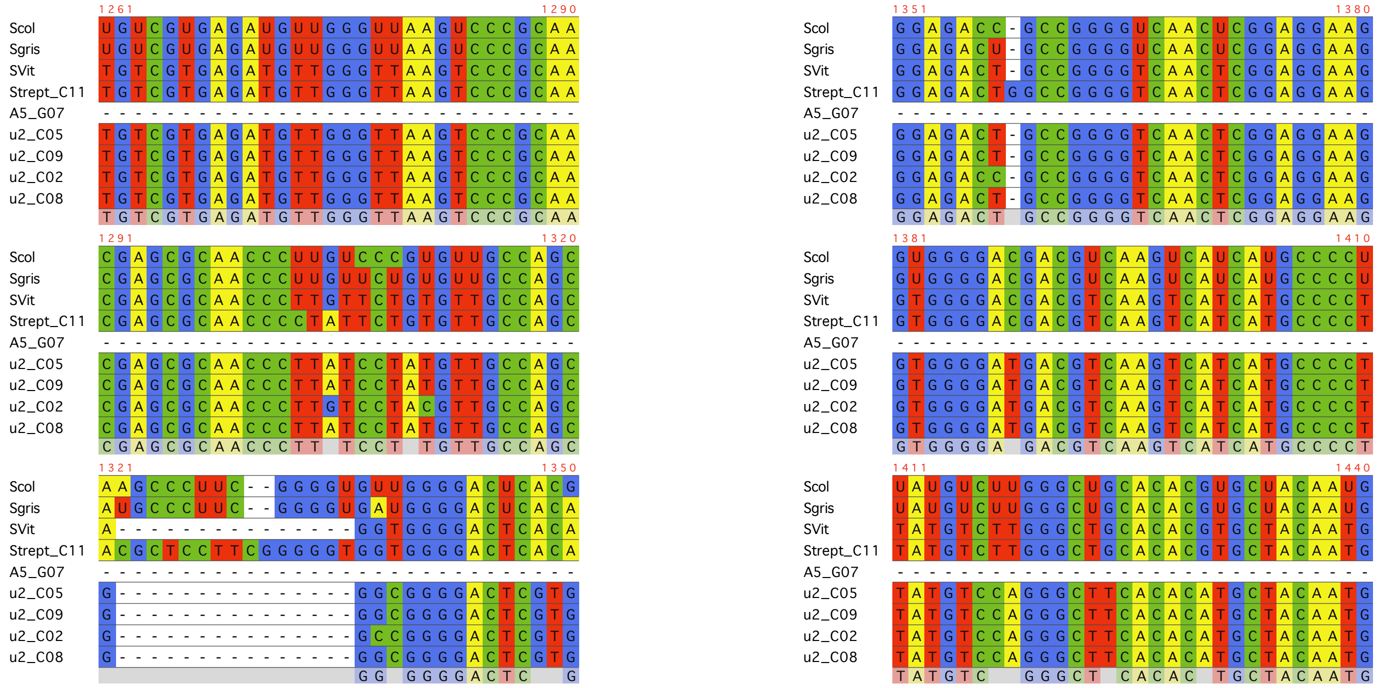
1261
1290
1291
1320
1321
1350
1351
1380
1381
1410
1411
1440
Scol
Sgris
SVit
Strept_C11
A5_G07
u2_C05
u2_C09
u2_C02
u2_C08

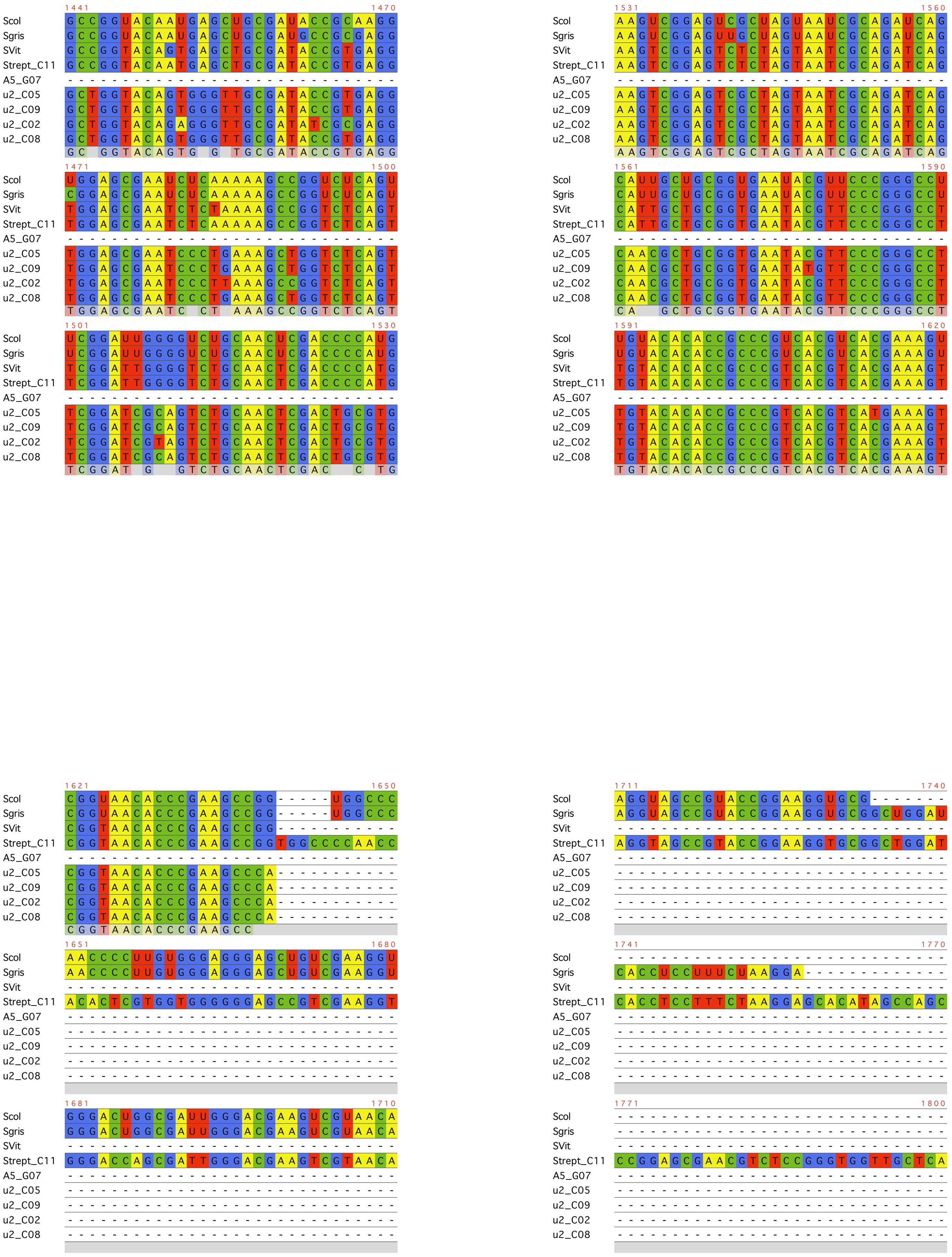
1441 1470
1471 1500
1501 1530
1531 1560
1561 1590
1591 1620
1621 1650
1651 1680
1681 1710
1711 1740
1741 1770
1771 1800
Scol
Sgris
SVit
Strept_C11
A5_G07
u2_C05
u2_C09
u2_C02
u2_C08

	1801 – 1830
Scol	------------------------------
Sgris	------------------------------
SVit	------------------------------
Strept_C11	TGGGTGGAACGTTGATTATTCGGCACCTGG
A5_G07	------------------------------
u2_C05	------------------------------
u2_C09	------------------------------
u2_C02	------------------------------
u2_C08	------------------------------

	1831 – 1860
Scol	------------------------------
Sgris	------------------------------
SVit	------------------------------
Strept_C11	TCACTGACGGTGACTGCTAGTACTGCACCC
A5_G07	------------------------------
u2_C05	------------------------------
u2_C09	------------------------------
u2_C02	------------------------------
u2_C08	------------------------------

	1861 – 1890
Scol	------------------------------
Sgris	------------------------------
SVit	------------------------------
Strept_C11	CCCTTCTTCGGGGGGTGCGTGGAACGCGGT
A5_G07	------------------------------
u2_C05	------------------------------
u2_C09	------------------------------
u2_C02	------------------------------
u2_C08	------------------------------

	1891 – 1920
Scol	------------------------------
Sgris	------------------------------
SVit	------------------------------
Strept_C11	TCGCTCTCGGGTCCGGGTGCCGGGCGCGCT
A5_G07	------------------------------
u2_C05	------------------------------
u2_C09	------------------------------
u2_C02	------------------------------
u2_C08	------------------------------

	1921 – 1950
Scol	------------------------------
Sgris	------------------------------
SVit	------------------------------
Strept_C11	GTTGGGTCCTGAGGGCACGGCCGTGAGTGT
A5_G07	------------------------------
u2_C05	------------------------------
u2_C09	------------------------------
u2_C02	------------------------------
u2_C08	------------------------------

	1951 – 1962
Scol	------------
Sgris	------------
SVit	------------
Strept_C11	CGCGGTTGGCCT
A5_G07	------------
u2_C05	------------
u2_C09	------------
u2_C02	------------
u2_C08	------------
