# Supplementary material for: No Ancient DNA Damage in Actinobacteria from the Neanderthal Bone
Source: PLoS One. 2013 May 3;8(5):e62799. doi: 10.1371/journal.pone.0062799 (PMC3643900; doi:10.1371/journal.pone.0062799)
Supplement: Table S1 — Statistics for the raw and pre-processed datasets. The raw reads of the Mammoth dataset contains the 4 bp adaptor sequence at the beginning of each read. The raw reads of the Neanderthal dataset contains the 4 bp adaptor sequence at the beginning of each read and a complete 44 bp, a partial or no adaptor sequence at the end of each read. (DOCX) [file pone.0062799.s008.docx]

**Table S1.**

|  | **Mammoth** | **Neandertal** | | **Neandertal** | **Neandertal** |
| --- | --- | --- | --- | --- | --- |
| restriction enzyme treatment | none | none | | Mix1 | Mix2 |
| Raw reads | 302,692 | 54,528,266 | | 30,725,159 | 84,993,767 |
| Raw Mb | 33.7^a^ | 7,222^b^ | | 3,032^b^ | 7,976^b^ |
| Clustering | cd-hit-454 | clustar | cd-hit-454 | cd-hit-454 | cd-hit-454 |
| Pre-processed reads | 203,353 | 39,480,490 | 48,920,467 | 27,326,674 | 69,636,377 |
| Pre-processed Mb | 21.2 | 3,949 | 4,827 | 1,988 | 4,754 |
