# Supplementary material for: No Ancient DNA Damage in Actinobacteria from the Neanderthal Bone
Source: PLoS One. 2013 May 3;8(5):e62799. doi: 10.1371/journal.pone.0062799 (PMC3643900; doi:10.1371/journal.pone.0062799)
Supplement: Table S2 — Clustering with clustar for each of the library emulsions in the untreated Neanderthal dataset. (DOCX) [file pone.0062799.s009.docx]

**Table S2.**

| emulsion | Sequencing runs | Identity level |
| --- | --- | --- |
| Vi80.P1 | NT118, NT119, NT124 | 91.43 |
| Vi80.P3.1 | NT189, NT190, NT191, NT192, NT194, NT211 | 89.93 |
| Vi80.P3.10 | NT247, NT248, NT249, NT250, NT268 | 89.91 |
| Vi80.P3.11 | NT251, NT252, NT253, NT254, NT255, NT256, NT257, NT299 | 89.77 |
| Vi80.P3.12 | NT258, NT259, NT260, NT261, NT265 | 89.77 |
| Vi80.P3.13 | NT279, NT280, NT281 | 89.71 |
| Vi80.P3.14 | NT282, NT283, NT284, NT286 | 90.10 |
| Vi80.P3.15 | NT285, NT287, NT288 | 89.81 |
| Vi80.P3.16 | NT289, NT290, NT291, NT292 | 89.93 |
| Vi80.P3.17 | NT293, NT294, NT295, NT296 | 89.91 |
| Vi80.P3.18 | NT297, NT298 | 89.94 |
| Vi80.P3.19 | NT315, NT316, NT317, NT318, NT319, NT320 | 89.57 |
| Vi80.P3.20 | NT321, NT322, NT323, NT324, NT325, NT326, NT394 | 89.78 |
| Vi80.P3.21 | NT327, NT328, NT330, NT331, NT332, NT395 | 89.86 |
| Vi80.P3.22 | NT333, NT334, NT335, NT329, NT344, NT345, NT359 | 90.08 |
| Vi80.P3.23 | NT346, NT347, NT348, NT350, NT358 | 89.99 |
| Vi80.P3.24 | NT351, NT352, NT353, NT362 | 90.01 |
| Vi80.P3.25 | NT354, NT355, NT356, NT357, NT367, NT392 | 89.49 |
| Vi80.P3.26 | NT360, NT361, NT363, NT364, NT365, NT366, NT368, NT369, NT370, NT371, NT372, NT393 | 89.70 |
| Vi80.P3.2 | NT193, NT195, NT196, NT209, NT210, NT212 | 89.81 |
| Vi80.P3.3 | NT213, NT214, NT215, NT216, NT217, NT218, NT262 | 89.98 |
| Vi80.P3.4 | NT219, NT220, NT221, NT222, NT223, NT224 | 89.80 |
| Vi80.P3.5 | NT225, NT226, NT266 | 89.84 |
| Vi80.P3.6 | NT227, NT228, NT229, NT230, NT263 | 90.02 |
| Vi80.P3.7 | NT231, NT232, NT233, NT234 | 89.95 |
| Vi80.P3.8 | NT236, NT237, NT238, NT239, NT240, NT241, NT267 | 89.68 |
| Vi80.P3.9 | NT235, NT242, NT243, NT244, NT245, NT246, NT264 | 89.83 |
| Vi80.P4.1 | NT184, NT181, NT180 | 88.80 |
| Vi80.P4.2 | NT179, NT182, NT183, NT185 | 88.76 |
| Vi80.P4.3 | NT186, NT187, NT188 | 88.81 |
