# Supplementary material for: No Ancient DNA Damage in Actinobacteria from the Neanderthal Bone
Source: PLoS One. 2013 May 3;8(5):e62799. doi: 10.1371/journal.pone.0062799 (PMC3643900; doi:10.1371/journal.pone.0062799)
Supplement: Table S3 — Performance tests of database searches. Performance of BLASTn searches using raw reads and reads in the assembly of the Bartonella bovis genome as queries against a database of more than 2,000 microbial genomes (P) and a cleaned up version of the SILVARef111 rRNA sequence database (eSILVA), respectively. Searches were performed against databases that included (P+ and eSILVA+) or excluded (P- and eSILVA-) related Bartonella sequences. (DOCX) [file pone.0062799.s010.docx]

**Table S3.**

| **Database** | **Query** | **E-value** | **Hits% (count)** | **Mean/Median** | ***Bartonella*/Alpha%** |
| --- | --- | --- | --- | --- | --- |
| P+ | Raw reads | 1e-3 | 61 | 2e-05/2e-15 | 99/99 |
| P- | Raw reads | 1e-3 | 12 | 1e-04/2e-06 | 0/47 |
| P- | Raw reads | 1e-10 | 2 | 9e-12/1e-14 | 0/63 |
| eSILVA+ | Assembly reads | 1e-3 | 0.046 (1337) | 4e-06/2e-52 | 89/98 |
| eSILVA- | Assembly reads | 1e-3 | 0.046 (1334) | 4e-06/6e-43 | 0/95 |
| eSILVA- | Assembly reads | 1e-10 | 0.044 (1276) | 5e-13/1e-43 | 0/96 |
