# Supplementary material for: No Ancient DNA Damage in Actinobacteria from the Neanderthal Bone
Source: PLoS One. 2013 May 3;8(5):e62799. doi: 10.1371/journal.pone.0062799 (PMC3643900; doi:10.1371/journal.pone.0062799)
Supplement: Table S4 — Precision and recall of searches against the eSILVA database. Reads in the assembly of the Bartonella bovis genome was used as queries with the e-value threshold set at e-10. The precision was calculated as TP/(TP+FP) and the recall as TP/(TP+FN). TP = True positives, calculated as the number of hits with > = 50 bp overlap with the SSU/LSU gene. FP = False positives, calculated as the number of reads showing hits that map outside the rRNA operons. FN = False negatives, calculated as true rRNA reads with no hits. SSU = Small Subunit rRNA; LSU = Large Subunit rRNA. (DOCX) [file pone.0062799.s011.docx]

**Table S4.**

|  | SSU count | SSU % | LSU count | LSU % |
| --- | --- | --- | --- | --- |
| Precision | 393 / (393+16) | 96 | 787 / (787+9) | 99 |
| Recall | 393 / ( 393+34) | 92 | 787 / (787+50) | 94 |
