# Supplementary material for: No Ancient DNA Damage in Actinobacteria from the Neanderthal Bone
Source: PLoS One. 2013 May 3;8(5):e62799. doi: 10.1371/journal.pone.0062799 (PMC3643900; doi:10.1371/journal.pone.0062799)
Supplement: Table S5 — Classification of the identified small and large subunit rRNA gene sequences in the Neanderthal dataset at the domain-level. Included in the analyses were the untreated and restriction enzyme treated (Mix1 and Mix2) datasets. (DOCX) [file pone.0062799.s012.docx]

**Table S5.**

|  | **untreated** | | | | **Mix1** | | **Mix2** | |
| --- | --- | --- | --- | --- | --- | --- | --- | --- |
| Clustering | clustar | | cd-hit-454 | | cd-hit-454 | | cd-hit-454 | |
| rRNA | LSU (%) | SSU (%) | LSU (%) | SSU (%) | LSU (%) | SSU (%) | LSU (%) | SSU (%) |
| all eukaryotes | 2666 (9) | 1708 (10) | 3201 (9) | 2098 (10) | 1632 (4) | 1205 (5) | 6010 (6) | 3411 (9) |
| Metazoa | 2417 (8) | 1608 (9) | 2914 (8) | 1971 (9) | 1310 (3) | 1080 (4) | 4550 (5) | 2928 (7) |
| Fungi | 62 (0) | 33 (0) | 67 (0) | 43 (0) | 58 (0) | 38 (0) | 207 (0) | 135 (0) |
| other eukaryotes | 187 (1) | 67 (0) | 220 (1) | 84 (0) | 264 (1) | 87 (0) | 1253 (1) | 348 (1) |
| Bacteria | 27440 (91) | 15674 (89) | 32614 (90) | 18880 (88) | 41425 (96) | 23165 (94) | 94113 (93) | 35758 (90) |
| all Archaea | 200 (1) | 318 (2) | 247 (1) | 408 (2) | 178 (0) | 253 (1) | 535 (1) | 623 (2) |
| Thaum-archaeota | 157 (1) | 313 (2) | 195 (1) | 403 (2) | 117 (0) | 243 (1) | 350 (0) | 592 (1) |
| other  Archaea | 43 (0) | 5 (0) | 52 (0) | 5 (0) | 61 (0) | 10 (0) | 185 (0) | 31 (0) |
| sum | 30306 (100) | 17700 (100) | 36062 (100) | 21386 (100) | 43235 (100) | 24623 (100) | 100658 (100) | 39792 (100) |
