# Supplementary material for: No Ancient DNA Damage in Actinobacteria from the Neanderthal Bone
Source: PLoS One. 2013 May 3;8(5):e62799. doi: 10.1371/journal.pone.0062799 (PMC3643900; doi:10.1371/journal.pone.0062799)
Supplement: Table S7 — Classification of the identified small and large subunit rRNA gene sequences in the Neanderthal dataset within the Actinobacteria. Included in the analyses were the untreated and restriction enzyme treated (Mix1 and Mix2) datasets. The category called “Actinobacteria” include groups other than those specified, as well as reads that could not be classified below the phylum level. (DOCX) [file pone.0062799.s014.docx]

**Table S7.**

|  | **untreated** | | | | **Mix1** | | **Mix2** | |
| --- | --- | --- | --- | --- | --- | --- | --- | --- |
| Clustering | clustar | | cd-hit-454 | | cd-hit-454 | | cd-hit-454 | |
| rRNA | LSU (%) | SSU (%) | LSU (%) | SSU (%) | LSU (%) | SSU (%) | LSU (%) | SSU (%) |
| Actinobacteria^a^ | 1808 (8) | 1085 (9) | 2185 (9) | 1328 (9) | 5495 (14) | 2523 (13) | 15527 (18) | 4184 (16) |
| Acidimicrobiales | 621 (3) | 1095 (9) | 751 (3) | 1305 (9) | 59 (0) | 325 (2) | 110 (0) | 813 (3) |
| Actinomycetales | 99 (0) | 28 (0) | 121 (0) | 39 (0) | 343 (1) | 128 (1) | 545 (1) | 250 (1) |
| Propionibacteriales | 3569 (17) | 2269 (19) | 4208 (17) | 2692 (19) | 7354 (19) | 5240 (27) | 15692 (18) | 5717 (21) |
| Corynebacteriales | 982 (5) | 530 (5) | 1181 (5) | 665 (5) | 2617 (7) | 1254 (6) | 8138 (9) | 2328 (9) |
| Frankiales | 1028 (5) | 54 (0) | 1229 (5) | 66 (0) | 2347 (6) | 275 (1) | 5152 (6) | 276 (1) |
| Pseudonocardiales | 808 (4) | 168 (1) | 937 (4) | 198 (1) | 2032 (5) | 606 (3) | 4283 (5) | 962 (4) |
| Glycomycetales | 250 (1) | 20 (0) | 299 (1) | 24 (0) | 152 (0) | 16 (0) | 249 (0) | 48 (0) |
| Micrococcales | 1723 (8) | 690 (6) | 2041 (8) | 819 (6) | 3728 (9) | 1420 (7) | 11443 (13) | 1541 (6) |
| Micromonosporales | 1564 (7) | 1066 (9) | 1854 (7) | 1281 (9) | 1671 (4) | 1269 (6) | 2083 (2) | 1436 (5) |
| Streptomycetales | 7484 (35) | 3875 (33) | 8870 (35) | 4651 (33) | 9946 (25) | 4414 (22) | 18012 (20) | 6007 (22) |
| Streptosporangiales | 1456 (7) | 779 (7) | 1750 (7) | 940 (7) | 3562 (9) | 2252 (11) | 7072 (8) | 3297 (12) |
| sum | 21392 (100) | 11659 (100) | 25426 (100) | 14008 (100) | 39306 (100) | 19722 (100) | 88306 (100) | 26859 (100) |
