# Supplementary material for: No Ancient DNA Damage in Actinobacteria from the Neanderthal Bone
Source: PLoS One. 2013 May 3;8(5):e62799. doi: 10.1371/journal.pone.0062799 (PMC3643900; doi:10.1371/journal.pone.0062799)
Supplement: Table S8 — List of the 454 library emulsions. Listed are the emulsions of two DNA extractions (extract and re-extract) and the sequencing runs corresponding to the extract (see table S2 for a list of sequencing runs for each emulsion set). (DOCX) [file pone.0062799.s015.docx]

**Table S8.**

| re-extract | Vi80.P1, Vi80.P3.1, Vi80.P3.2, Vi80.P3.3, Vi80.P3.4, Vi80.P3.5, Vi80.P3.6, Vi80.P3.7, Vi80.P3.8 , Vi80.P3.9, Vi80.P3.10, Vi80.P3.11, Vi80.P3.12, Vi80.P3.13, Vi80.P3.14, Vi80.P3.15, Vi80.P3.16, Vi80.P3.17, Vi80.P3.18, Vi80.P3.19, Vi80.P3.20, Vi80.P3.21, Vi80.P3.22, Vi80.P3.23 , Vi80.P3.24, Vi80.P3.25, Vi80.P3.26 |
| --- | --- |
| extract | Vi80.P4.1 (NT184, NT181, NT180), Vi80.P4.2 (NT179, NT182, NT183, NT185), Vi80.P4.3 (NT186, NT187, NT188) |
