# Supplementary material for: No Ancient DNA Damage in Actinobacteria from the Neanderthal Bone
Source: PLoS One. 2013 May 3;8(5):e62799. doi: 10.1371/journal.pone.0062799 (PMC3643900; doi:10.1371/journal.pone.0062799)
Supplement: Table S9 — Classification of the identified rRNA gene sequences in the DNA extracted from the cave sediment sample and in the Mammoth dataset at the phylum-level. Universal primers (27f, 1492r) were used for the PCR amplifications of the small subunit rRNA gene sequences from the cave sediment sample. The category called “Bacteria” include phyla other than those specified, as well as reads that could not be classified below the domain level. The number of identified sequences is shown, with the percent given in parenthesis. (DOCX) [file pone.0062799.s016.docx]

**Table S9.**

|  | Sediment SSU clones^a^(%) | Mammoth rRNA reads (%) |
| --- | --- | --- |
| Acidobacteria | 2 (1) | 3 (3) |
| Actinobacteria | 231 (83) | 23 (27) |
| Bacteroidetes | 0 (0) | 14 (16) |
| Firmicutes | 2 (1) | 10 (12) |
| Planctomycetes | 0 (0) | 0 (0) |
| Proteobacteria | 26 (9) | 27 (31) |
| Verrucomicrobia | 0 (0) | 0 (0) |
| Bacteria | 2 (1) | 9 (10) |
