# Supplementary material for: No Ancient DNA Damage in Actinobacteria from the Neanderthal Bone
Source: PLoS One. 2013 May 3;8(5):e62799. doi: 10.1371/journal.pone.0062799 (PMC3643900; doi:10.1371/journal.pone.0062799)
Supplement: Table S10 — Classification of the identified rRNA gene sequences in the DNA extracted from the cave sediment and in the Mammoth dataset within the Actinobacteria. The number of identified sequences is shown, with the percent given in parenthesis. (DOCX) [file pone.0062799.s017.docx]

**Table S10.**

|  | Sediment SSU clones (%) | Mammoth rRNA reads (%) |
| --- | --- | --- |
| Actinobacteria | 40 (13) | 4 (17) |
| Acidimicrobiales | 31 (10) | 0 (0) |
| Actinomycetales | 0 (0) | 0 (0) |
| Propionibacteriales | 99 (31) | 1 (4) |
| Corynebacteriales | 0 (0) | 1 (4) |
| Frankiales | 0 (0) | 0 (0) |
| Pseudonocardiales | 128 (40) | 0 (0) |
| Glycomycetales | 7 (2) | 0 (0) |
| Micrococcales | 12 (4) | 17 (74) |
| Micromonosporales | 0 (0) | 0 (0) |
| Streptomycetales | 1 (0) | 0 (0) |
| Streptosporangiales | 0 (0) | 0 (0) |

.
