# Supplementary material for: No Ancient DNA Damage in Actinobacteria from the Neanderthal Bone
Source: PLoS One. 2013 May 3;8(5):e62799. doi: 10.1371/journal.pone.0062799 (PMC3643900; doi:10.1371/journal.pone.0062799)
Supplement: Table S11 — The most abundant MEROPS protease families identified in the Neanderthal dataset. Family type peptidase refer to the protein that is the representative of the whole family. The ten most abundant MEROPS protease families were chosen from the list of MEROPS identities that had at least 0.5% abundance. The abundance was calculated from the number of hits to each of the families in a BLASTx search of all reads against the MEROPS peptidase protein sequence database (e-value threshold 10−10). The biological function description was taken directly from the MEROPS website. (DOCX) [file pone.0062799.s018.docx]

**Table S11.**

| Family type peptidase | | |
| --- | --- | --- |
| MEROPS ID | abundance | Peptidases and Homologues |
| Biological functions | | |
| S33.001 - prolyl aminopeptidase (Neisseria gonorrhoeae), MEROPS Accession MER000431 (peptidase unit: 1-310) | | |
| S33.UNW | 6.3% | family S33 non-peptidase homologues |
| S33.UPW | 5.7% | family S33 unassigned peptidases |
| Most members of the family are synthesized with signal peptides and are thus either secreted or periplasmic enzymes. Prolyl aminopeptidase activity is not essential for bacterial growth, but may confer a selective advantage allowing a bacterium to utilize proline-rich substrates. | | |
| M38.001 - isoaspartyl dipeptidase (metallo-type) (Escherichia coli), MEROPS Accession MER001495 (peptidase unit: 10-359) | | |
| M38.UNW | 7.6% | family M38 non-peptidase homologues |
| M38.UPW | 0.8% | family M38 unassigned peptidases |
| The beta-aspartyl dipeptidase is a cytosolic enzyme and is a product of the iadA gene in Escherichia coli. Its function is the release of isoaspartate residues from peptides which accumulate during the stationery phase of bacterial growth and may be toxic. However, isoaspartates do not accumulate in iadA knockouts, probably because another enzyme is capable of releasing them from peptides (Gary & Clarke, 1995). Isoaspartates can also be metabolized by L-asparaginase (T02.002, Kelo et al., 2002), which also hydrolyses asparagine, though kinetic analysis suggests that the isoaspartyl dipeptidase activity is the major activity (Borek et al., 2004. The structure of L-asparaginase has been determined, showing that it is a N-terminal nucleophile hydrolase, unrelated to enzymes in family M38 (Prahl et al., 2004), which processes itself by cleavage of a normal peptide bond (Hejazi et al., 2002). | | |
| S09.001 - prolyl oligopeptidase (Sus scrofa), MEROPS Accession MER000392 (peptidase unit: 428-710) | | |
| S09.UNW | 3.3% | family S9 non-peptidase homologues |
| S09.UPW | 3.4% | family S9 unassigned peptidases |
| Many peptidases in the family are believed to be important for the degradation of biologically active peptides: for example, DPP-IV metabolizes the insulinotropic hormone, glucagon-like peptide 1 (Holst & Deacon, 1998; Deacon & Holst, 2002). Members of the family are found in different cellular locations: prolyl oligopeptidase in intracellular, DDP-IV and fibroblast activation protein α (S09.007) are expressed on cell surfaces; and oligopeptidase B is secreted to the bacterial periplasm (Tsuru, 1998). | | |
| S08.001 - subtilisin Carlsberg (Bacillus licheniformis), MEROPS Accession MER000309 (peptidase unit: 106-379) | | |
| S08.069 | 0.8% | SAM-P45 peptidase |
| S08.UPA | 4.6% | subfamily S8A unassigned peptidases |
| In bacteria, archaea and fungi, family members are probably involved in nutrition. Most are secreted, but lactocepin (S08.018) is attached to the outer surface of the cell wall, and lantibiotic leader peptidases are intracellular. Lantibiotic leader peptidases, such as the CylP leader peptidase (S08.086), are required for the activation of lantibiotics and cytolysins. Subfamily S8A peptidases from Dichelobacter have been implicated in the pathogenesis of ovine foot-rot (Kortt & Stewart, 2004) and C5a peptidase (S08.020) from pathogenic streptococci destroys the complement chemotaxin C5a (Cleary & Matsuka, 2004). | | |
| M20.001 - glutamate carboxypeptidase (Pseudomonas sp.), MEROPS Accession MER001266 (peptidase unit: 23-415) | | |
| M20.UNW | 0.6% | family M20 non-peptidase homologues |
| M20.UPA | 2.6% | subfamily M20A unassigned peptidases |
| M20.UPD | 0.7% | family M20D unassigned peptidases |
| In general, the peptidases hydrolyse the late products of protein degradation so as to complete the conversion of proteins to free amino acids. The Pseudomonas glutamate carboxypeptidase is a periplasmic enzyme that is synthesised with a signal peptide. | | |
| M23.001 - beta-lytic metallopeptidase (Achromobacter lyticus), MEROPS Accession MER001281 (peptidase unit: 196-374) | | |
| M23.UNB | 2.0% | subfamily M23B non-peptidase homologues |
| M23.UPB | 0.9% | subfamily M23B unassigned peptidases |
| The methionyl aminopeptidases of subfamily M24A are essential for the removal of the initiating methionine of many proteins, acting co-translationally in association with the ribosomes (Chang et al., 1992). The X-Pro dipeptidase found in eukaryotes has a role in the cleavage of XaaPro linkages found in dipeptides associated with collagen recycling. Deficiency results in an increase of these dipeptides to toxic levels (Myara et al., 1994). | | |
| C44.001 - amidophosphoribosyltransferase precursor (Homo sapiens), MEROPS Accession MER003314 (peptidase unit: 12-277) | | |
| C44.001 | 0.6% | amidophosphoribosyltransferase precursor |
| C44.UNW | 0.7% | family C44 non-peptidase homologues |
| C44.UPW | 1.3% | family C44 unassigned peptidases |
| Evidently the autolytic processing of the precursor of amidophosphoribosyltransferase makes available the catalytic activity of the transferase, but the function of the latency of the precursor is not understood, since several homologous enzymes with similar kinds of activity are synthesised already in active form. | | |
| M06.001 - InhA1 peptidase (Bacillus thuringiensis), MEROPS Accession MER001164 (peptidase unit: 41-687) | | |
| M06.UPW | 2.2% | family M6 unassigned peptidases |
| Family M6 contains a metalloendopeptidase from Bacillus thuringiensis known as immune inhibitor A. This contributes to the virulence of the bacterium, degrading the defensive cecropins and attacin of the immune system of the lepidopteran insects it infects. | | |
| M24.001 - methionyl aminopeptidase 1 (Escherichia coli), MEROPS Accession MER001243 (peptidase unit: 1-264) | | |
| M24.001 | 1.1% | methionyl aminopeptidase 1 |
| M24.UNB | 0.9% | subfamily M24B non-peptidase homologues |
| The methionyl aminopeptidases of subfamily M24A are essential for the removal of the initiating methionine of many proteins, acting co-translationally in association with the ribosomes (Chang et al., 1992). The X-Pro dipeptidase found in eukaryotes has a role in the cleavage of XaaPro linkages found in dipeptides associated with collagen recycling. Deficiency results in an increase of these dipeptides to toxic levels (Myara et al., 1994). | | |
| C26.001 - gamma-glutamyl hydrolase (Rattus norvegicus), MEROPS Accession MER002464 (peptidase unit: 25-293) | | |
| C26.UNW | 0.9% | family C26 non-peptidase homologues |
| C26.UPW | 1% | family C26 unassigned peptidases |
| Gamma-glutamyl hydrolase is a lysosomal enzyme probably involved in the turnover of folyl poly-gamma-glutamates. | | |
| M09.001 - bacterial collagenase V (Vibrio alginolyticus), MEROPS Accession MER001172 (peptidase unit: 76-531) | | |
| M09.001 | 0.4% | bacterial collagenase V |
| Both the Vibrio and Clostridium collagenases are secreted enzymes, and are synthesized as precursors. Infection by Clostridium histolyticum is a cause of gas gangrene. | | |
