# Supplementary material for: No Ancient DNA Damage in Actinobacteria from the Neanderthal Bone
Source: PLoS One. 2013 May 3;8(5):e62799. doi: 10.1371/journal.pone.0062799 (PMC3643900; doi:10.1371/journal.pone.0062799)
Supplement: Table S12 — Statistics for the assembled contigs of the rRNA, collagenase and aminopeptidase genes putatively assigned to Streptomyces. The relative gene copy numbers are based on the assumption that that the collagenase and aminopeptidase are singly-copy genes, while the rRNA genes are present in six copies per genome. (DOCX) [file pone.0062799.s019.docx]

**Table S12.**

|  | length | reads | total bp | coverage | gene copy numbers | | |
| --- | --- | --- | --- | --- | --- | --- | --- |
| Collagenase Contig 113 | 2,947 | 789 | 121,962 | 41 | 1.00 | 1.21 | 1.58 |
| SSU_Streptomycinae C11 | 2,556 | 2,754 | 529,567 | 207 | 5.05 | 6.09 | 7.96 |
| LSU_Streptomycinae C35 | 4,667 | 4,681 | 948,149 | 203 | 4.95 | 5.97 | 7.81 |
| Aminopeptidase Contig C1104 | 1,607 | 225 | 41,978 | 26 | 0.63 | 0.76 | 1.00 |
