# Supplementary material for: No Ancient DNA Damage in Actinobacteria from the Neanderthal Bone
Source: PLoS One. 2013 May 3;8(5):e62799. doi: 10.1371/journal.pone.0062799 (PMC3643900; doi:10.1371/journal.pone.0062799)
Supplement: Table S13 — Comparison of the fraction of rRNA and total bacterial gene sequences in the Neanderthal datasets. The threshold for the identification of the rRNA genes was set to an e-value of 100. The dataset in the upper row was clustered with cluster, while the three other datasets were clustered with cd-hit-454. (DOCX) [file pone.0062799.s020.docx]

**Table S13.**

| Treatment | Total Mb | Streptomycineae fraction x10^-4^ (bp) | Bacteria fraction x10^-4^ (bp) |
| --- | --- | --- | --- |
| None | 3,949 | 3.42 (1,352,019) | 12.20 (4,815,965) |
| None | 4,827 | 3.30 (1,592,700) | 11.71 (5,653,538) |
| Mix1 | 1,988 | 4.79 (952,852) | 19.75 (3,927,119) |
| Mix2 | 4,754 | 1.70 (807,787) | 8.91 (4,237,333) |
