# Supplementary material for: No Ancient DNA Damage in Actinobacteria from the Neanderthal Bone
Source: PLoS One. 2013 May 3;8(5):e62799. doi: 10.1371/journal.pone.0062799 (PMC3643900; doi:10.1371/journal.pone.0062799)
Supplement: Table S14 — Mean lengths of genomic fragments after DNA digestions with restriction enzymes in silico. The restriction sites were mapped to the genomes with patman. Several Streptomyces genomes as well as a few other genomes of lower GC was included in the analysis. (DOCX) [file pone.0062799.s021.docx]

**Table S14.**

|  | GC | Mix2 | Mix1 |
| --- | --- | --- | --- |
| Pseudonocardia dioxanivorans | 73.1 | 11 | 21 |
| Streptomyces cattleya | 73.0 | 13 | 34 |
| Streptomyces griseus | 72.2 | 14 | 32 |
| Streptomyces coelicolor | 72.0 | 14 | 31 |
| Streptomyces hygroscopicus jinggangensis | 71.8 | 14 | 33 |
| Streptomyces SirexAA | 71.7 | 14 | 33 |
| Streptomyces scabiei | 71.5 | 14 | 33 |
| Streptomyces flavogriseus | 71.0 | 15 | 33 |
| Streptomyces violaceusniger | 70.9 | 14 | 36 |
| Streptosporangium roseum | 70.9 | 14 | 35 |
| Streptomyces bingchenggensis | 70.8 | 14 | 34 |
| Streptomyces avermitilis | 70.7 | 14 | 32 |
| Intrasporangium calvum | 70.7 | 15 | 30 |
| Propionibacterium acnes | 60.0 | 30 | 62 |
| Corynebacterium glutamicum | 54.1 | 40 | 103 |
| Escherichia coli | 50.0 | 37 | 108 |
