# Supplementary material for: No Ancient DNA Damage in Actinobacteria from the Neanderthal Bone
Source: PLoS One. 2013 May 3;8(5):e62799. doi: 10.1371/journal.pone.0062799 (PMC3643900; doi:10.1371/journal.pone.0062799)
Supplement: Table S15 — List of sequences containing rRNAs in the truncated SILVA database, as detected by tRNAscan-SE. (DOCX) [file pone.0062799.s022.docx]

**Table S15.**

| Accession | tRNA Begin | Bounds End | tRNA Type | Cove Score | cut-head | cut-tail | alignment-quality | pintail |
| --- | --- | --- | --- | --- | --- | --- | --- | --- |
| Database: LSURef_111_tax_silva_trunc | | | | | | | | |
| AASG02005286.11698.14562 | 2781 | 2854 | Arg | 51.16 | 0 | 0 | 52 | - |
| AASG02001643.3.2889 | 565 | 648 | Tyr | 44.67 | 0 | 0 | 65 | - |
| AEWC01031912.3857.6444 | 130 | 203 | His | 66.37 | 0 | 0 | 76 | - |
| AGQN01058016.752.3941 | 218 | 289 | Asp | 61.75 | 0 | 0 | 47 | - |
| CACX01001889.215528.217894 | 247 | 175 | Thr | 78.61 | 0 | 0 | 77 | - |
| HE663493.40751.43572 | 557 | 484 | Ile | 78.39 | 0 | 0 | 62 | - |
| Database: SSURef_111_NR_tax_silva_trunc | | | | | | | | |
| AFSQ01027585.1790.3265 | 95 | 166 | Val | 66.47 | 0 | 0 | 68 | 0 |
| AM990992.2181279.2182758 | 4 | 86 | Leu | 57.20 | 0 | 0 | 85 | - |
| AM990992.2181279.2182758 | 108 | 178 | Gly | 71.34 | 0 | 0 | 85 | - |
