# Supplementary material for: No Ancient DNA Damage in Actinobacteria from the Neanderthal Bone
Source: PLoS One. 2013 May 3;8(5):e62799. doi: 10.1371/journal.pone.0062799 (PMC3643900; doi:10.1371/journal.pone.0062799)
Supplement: Table S16 — List of scripts and analysis files available in the github repository. https://github.com/kasiazar/NeandertalBoneMetagenome. (DOCX) [file pone.0062799.s023.docx]

**Table S16.** List of scripts and analysis files available in the github repository <https://github.com/kasiazar/NeandertalBoneMetagenome>

| Location in the repository | File name | Description |
| --- | --- | --- |
| eSILVA | eSilvaRef111NR.fasta | Custom good quality rRNA database |
|  | Make_eSILVA.sh | Wrapper describing how to create the database |
|  | Silva_Cut_QualityFilter.pl | Script to remove poor quality |
|  | Silva_Ref111_Filter.pl | Script to remove poor taxonomy |
| Assembly | consed_SSU_Metazoa.tgz | SSU Neandertal - files for viewing in consed; intermediate substitution calculation files. |
|  | consed_LSU_Metazoa.tgz | LSU Neandertal - files for viewing in consed; intermediate substitution calculation files. |
|  | consed_SSU_Streptomyces .tgz | SSU Streptomyces - files for viewing in consed; intermediate substitution calculation files. |
| Assembly/SSU/ Streptomyces | Streptomycineae.C11.ace.1.details.ReadsDiscrepantSummerize_end5_rm5.all_reads | List of all reads that pass the cleaning procedure (381). |
| Assembly/SSU/ Streptomyces | Streptomycineae.C11.ace.1.details.ReadsDiscrepantSummerize_end5_rm5.skipped_too_many_substitutions | List of reads skipped due to too many (>5) substituions (21). |
|  | consed_LSU_Streptomyces .tgz | LSU Streptomyces - files for viewing in consed; intermediate substitution calculation files. |
| ContigsHtml | ContigsHtml_v1.tgz | folder packed |
|  | ContigsList.html | Html page listing all the contigs, start here. |
|  | [LS]SU*end0.html | Assemblies, every position disagreeing with the consensus in marked in black. |
|  | LS]SU*end5.rm5.html | Script calculating subsitutions produces with file as well for manual inspection. Every base that was calculated in marked in black. |
| Alignments | Pseudonocardia2_blocks2* | Alignment fasta file for Pseudonocardia (Figure S2) and names conversion file between the tree and the alignment. |
|  | Propionibacteria2_blocks1* | Alignment fasta file for Propionibacteria (Figure S3) and names conversion file between the tree and the alignment. |
|  | Streptomyces3_blocks3* | Alignment fasta file for Streptomyces (main Figure 4) and names conversion file between the tree and the alignment. |
